# Identification METTL18 as a Potential Prognosis Biomarker and Associated With Immune Infiltrates in Hepatocellular Carcinoma

**DOI:** 10.3389/fonc.2021.665192

**Published:** 2021-05-26

**Authors:** Tian-Hao Li, Cheng Qin, Bang-Bo Zhao, Hong-Tao Cao, Xiao-Ying Yang, Yuan-Yang Wang, Ze-Ru Li, Xing-Tong Zhou, Wei-Bin Wang

**Affiliations:** ^1^ Department, State Key Laboratory of Complex Severe and Rare Diseases, Peking Union Medical College Hospital, Chinese Academy of Medical Science and Peking Union Medical College, Beijing, China; ^2^ Department of General Surgery, Peking Union Medical College Hospital, Chinese Academy of Medical Science and Peking Union Medical College, Beijing, China; ^3^ Department of Breast Surgery, Peking Union Medical College Hospital, Chinese Academy of Medical Science and Peking Union Medical College, Beijing, China

**Keywords:** methyltransferase-like 18, hepatocellular carcinoma, RNA methylation, immune infiltration, nomogram

## Abstract

Methyltransferase-like 18 (METTL18), a METTL family member, is abundant in hepatocellular carcinoma (HCC). Studies have indicated the METTL family could regulate the progress of diverse malignancies while the role of METTL18 in HCC remains unclear. Data of HCC patients were acquired from the cancer genome atlas (TCGA) and gene expression omnibus (GEO). The expression level of METTL18 in HCC patients was compared with normal liver tissues by Wilcoxon test. Then, the logistic analysis was used to estimate the correlation between METTL18 and clinicopathological factors. Besides, Gene Ontology (GO), Gene Set Enrichment Analysis (GSEA), and single-sample Gene Set Enrichment Analysis (ssGSEA) were used to explore relevant functions and quantify the degree of immune infiltration for METTL18. Univariate and Multivariate Cox analyses and Kaplan–Meier analysis were used to estimate the association between METTL18 and prognosis. Besides, by cox multivariate analysis, a nomogram was conducted to forecast the influence of METTL18 on survival rates. METTL18-high was associated with Histologic grade, T stage, Pathologic stage, BMI, Adjacent hepatic tissue inflammation, AFP, Vascular invasion, and TP53 status (P < 0.05). HCC patients with METTL18-high had a poor Overall-Survival [OS; hazard ratio (HR): 1.87, P < 0.001), Disease-Specific Survival (DSS, HR: 1.76, P = 0.015), and Progression-Free Interval (PFI, HR: 1.51, P = 0.006). Multivariate analysis demonstrated that METTL18 was an independent factor for OS (HR: 2.093, P < 0.001), DSS (HR: 2.404, P = 0.015), and PFI (HR: 1.133, P = 0.006). Based on multivariate analysis, the calibration plots and C-indexes of nomograms showed an efficacious predictive effect for HCC patients. GSEA demonstrated that METTL18-high could activate G2M checkpoint, E2F targets, KRAS signaling pathway, and Mitotic Spindle. There was a positive association between the METTL18 and abundance of innate immunocytes (T helper 2 cells) and a negative relation to the abundance of adaptive immunocytes (Dendritic cells, Cytotoxic cells *etc.*). Finally, we uncovered knockdown of METTL18 significantly suppressed the proliferation, invasion, and migration of HCC cells *in vitro*. This research indicates that METTL18 could be a novel biomarker to evaluate HCC patients’ prognosis and an important regulator of immune responses in HCC.

## Introduction

Hepatocellular Carcinoma (HCC) is the fourth most common cause of death of tumor and the seventh most common malignant tumor in 2018 (1). Due to the hepatitis related virus being endemic, the majority of new patients of HCC occur in China annually (2). Most of HCC patients usually lost the opportunity of surgical treatment for the lack of specific symptoms. The molecular mechanisms of liver cancer initiation and progression are still unclear, which make it hard to attain effective treatment (3). In addition, problems remain in the process of treatment and diagnosis of liver cancer, due to the shortage of efficient biomarkers for cancer type and disease stage. In recent years, several serum biomarkers were used to detect the progression and prognosis of hepatocellular carcinoma, such as Alpha-fetoprotein (AFP) and Carcinoembryonic Antigen (CEA) (4, 5). However, both the specificity and sensitivity of these biomarkers in evaluating tumor progression, prognosis, and recurrence are still unsatisfactory. Therefore, identification of the reliable and new biomarkers for the diagnosis of HCC is urgently needed to improve the prognosis.

Methyltransferases contains a S-Adenosyl Methionine (SAM) binding domain, known as a family with a structurally conserved methyltransferase similar domains (6–9). Research studies have shown that methylation could influence chromatin organization and directly regulate transcription of gene, but modulating the mutations in the gene itself (8, 10). In addition, studies have demonstrated that methyltransferases could influence the progression of metabolic diseases, genetic diseases, and cancers (11–13). Studies have shown that members of the METTL family are involved in a variety of biological functions. For example, METTL2B, METTL3, METTL8, and METTL16 have been shown to be RNA methyltransferases (14, 15) and play pivotal roles in tumorigenesis (16). In addition, studies have also shown that the METTL family could play a key role in HCC. Chen et al. have demonstrated that METTL3 could promote the progression of liver cancer, and Ma et al. have indicated the metastatic ability of HCC could be suppressed by METTL14 (17, 18). However, the influence of other members of the METTL family in progression of hepatocellular carcinoma has not been thoroughly studied.

Methyltransferase-like 18, also known as METTL18, has been regarded as a protein-coding gene (19). A recent research indicated that METTL18 is an effective candidate protein for patients suffering Fibromyalgia syndrome (5). UniProt, the most authoritative protein database demonstrates that METTL18 performs as a methyltransferase, participating in modification of essential protein and process of binding heat shock protein (20). Nevertheless, little is known about METTL18 in cancer.

To better analyze the role of METTL18 in progression of HCC, we applied RNA-seq data from the TCGA and GEO datasets, with statistical and bioinformatics ways, such as differentially expressed genes (DEG) analysis, Kaplan–Meier (KM) survival analysis, and Cox & Logistic regression analysis, nomogram, Gene Ontology (GO) analysis, Gene Set Enrichment Analysis (GSEA), single-sample Gene Set Enrichment Analysis (ssGSEA). Moreover, we further knock down the expression of METTL18 *in vitro* to detect the impact on the ability of proliferation, invasion, and migration of hepatocellular carcinoma cells.

## Materials and Methods

### Data and Preprocessing

Expression data of RNA with clinical information from HCC patients (included 371 tumor and 50 normal tissues) were acquired from TCGA (https://portal.gdc.cancer.gov/); in addition, 225 tumor and 220 normal samples were acquired from GSE14520. The criteria of exclusion were OS less than 30 days and normal HCC tissues. Then, HTSeq-FPKM information of level 3 has been transformed into Transcripts Per Million (TPM); then TPM information of 371 HCC samples was applied for the next analyses. Unknown and unavailable clinical factors in 371 samples were deemed as missing values, and the information was demonstrated in **Supplementary Table 1**.

### Expression of METTL18 in HCC Samples in the TCGA and GEO Dataset

Applying disease state (normal or tumor) as variable, scatter plots and boxplots were performed to estimate different expression levels of METTL18. Using receiver operating characteristic (ROC) curves, we evaluated the diagnostic performance of METTL18 in HCC patients. By statistical ranking, expression level of METTL18 below or above the median was determined as METTL18-high or METTL18-low.

### Identification of DEGs Between METTL18-Low and -High Expression HCC Groups

Applying the Student’s t-test, DEGs between METTL18-low and METTL18-high samples from TCGA database were analyzed by DESeq2 (4.0) package. With the criteria of absolute log (FC) higher than 1.5 and the adj P-value <0.05, genes were deemed to be statistically positive. Then, DEGs were shown in the volcano plots and heat map.

### Enrichment of Biofunction and Infiltration of Immune Related Cell Evaluation

Metascape (http://metasape.org) was applied to explore the enrichment of METTL18 associated DEGs by pathway and process. Criteria contained: the enrichment factor >1.5, a minimum count of three, and P <0.01 to attain significantly statistically significance. Using GSEA, we have investigated the differences in pathways of biofunction between the METTL18-low and -high patients to analysis METTL18-associated pathways and phenotypes. With 1,000 times, the permutation test was applied to analyze significantly signal pathways. FDR <0.25 and adjusted P <0.01 were recognized as significantly associated genes. Graphical plots and statistically analysis were performed by R package ClusterProfiler (4.0) (21). Using STRING database, the protein–protein interaction (PPI) network was built by the DEGs (22), and the PPI pairs were selected with an interaction score >0.7. To better explore the tumor infiltration levels of immune cells, ssGSEA, interrogating expression data of genes in published gene lists (23), was used to quantify relative infiltration levels of 24 types of immune cell. Comprising 509 genes, the signatures applied containing a multiple set of innate as well as adaptive immune relative cell types (23). To evaluate the association between the infiltration levels of immune cells and METTL18 and the correlation of the different groups of METTL18 expression with infiltration of immune cells, Spearman correlation and Wilcoxon test were used.

### Clinical Analysis on Prognostic State, Model Construction and Estimation

Using R package (V3.6.2), we analyzed connection between clinicopathological characteristics and METTL18 by logistic regression and Wilcoxon signed-rank sum test. By Kaplan–Meier method and Cox regression, we analyzed the clinical pathologic factors related to 10-year overall survival (OS), disease-specific survival (DSS), and progression-free interval (PFI) in TCGA samples. Multivariate Cox analysis was applied to analyze the influence of METTL18 expression on prognosis along with other clinicopathologic factors (TNM stage, Histologic grade, Vascular invasion, Residual tumor, Albumin, AFP, TP53 status, Race, Child-Pugh grade, Adjacent hepatic tissue inflammation, Age, Gender, and Prothrombin time). Median of METTL18 expression level was chosen as the cut-off value. In all tests, P < 0.05 was defined statistically significant. Using KM method with a log-rank test, the difference of OS, DSS, as well as PFI between METTL18-low and -high group was analyzed. Acquired from multivariate Cox analysis, we used the independent prognostic factors to construct nomograms, evaluating the prognosis for 1, 3-, and 5-year, respectively. Applying the RMS package (https://cran.r-project.org/web/packages/rms/index.html), we established nomograms that included calibration plots and significant clinical factors. By drafting the nomogram evaluating probability against actually occurrence, the calibration curves were graphical evaluated, and the 45 degrees line indicated the best predictive values. Analyzed by bootstrap way with 1,000 resamples, a concordance index (C-index) was analyzed and applied to estimate the discrimination of the model. The separate prognostic factors and predictive accuracies of the nomogram were evaluated by the C-index. The statistical test was two tailed with a statistically significant level set at 0.05 in our research.

### Cell Culture and Transfection

Hepatocellular cancer cell lines (HepG2, M97H, LM3, Bel7402, SK-HEP1, and Huh7) were acquired from the American Type Culture Collection (Virginia, USA). They were cultured in Dulbecco’s Modified Eagle Medium (Hyclone) with 10% fetal bovine serum (Gibco) under incubation at 37°C with 5% carbon dioxide. Transfections were performed applying OPTI-MEM (Invitrogen) and Lipofectamine 3000 by the manufacturer’s instructions. The siMETTL18 (5′-GACTTTCCTTAGACTGTTA-3′) and siNC were bought from RiboBio (Guangzhou, China) and introduced into cells at a concentration of 50 nM. The transfected cells were harvested at 48 h after transfection.

### Western Blotting

Huh7, LM3 cells were seeded and cultured until the confluence reached 70%. Western Blotting (WB) was performed by the protocols of a previous research. Using a 10% gel, amounts of protein (25 µg) were subjected to Sodium Dodecyl Sulfate-Polyacrylamide Gel Electrophoresis (SDS-PAGE). The primary antibodies applied for the WB experiment were bought from Proteintech Group (Rosemont, United States): anti-METTL18 (Catalog number: 25553-1-AP; 1:1000), anti-GAPDH (Catalog number: 10494-1-AP; 1:5000) antibodies and antialpha tubulin (Catalog number: 11224-1-AP; 1:5,000) antibodies.

### Cell Proliferation, Invasion, and Migration Assays

The Cell Counting Kit-8 (CCK-8) and colony formation assays were applied to explore the ability of proliferation of cancer cells in different groups. In CCK-8 experiment, a total of 2,500 cancer cells were added into each well of 96-well plate. 10 µl of CCK-8 solution (Beijing Solarbio Science & Technology Co., Ltd, Bei, China) was added into 96-well, then the absorbance of each well was analyzed at 450 nm after an incubation at 37°C for 2 h. For colony formation experiment, 1,000 cells of different groups were added into each well of a six-well plate. The culture medium was changed every 72 h. Crystal violet and 4% paraformaldehyde were applied to stain and fix the cells when the appearance of colonies could be recognized. The wound healing and transwell assays were applied to explore the ability of cellular migration and invasion.

### Statistical Analysis

Statistical analyses were performed by RStudio software and the R software (version 3.8.0). One-way analysis of variance (ANOVA) and two tailed Student’s t-test were applied to analyzed the data. Statistical significance of the difference was set at *P*–value <0.05.

## Results

### Expression Level of METTL18 in Pan-Cancers and Hepatocellular Carcinoma

**Figure 1:**
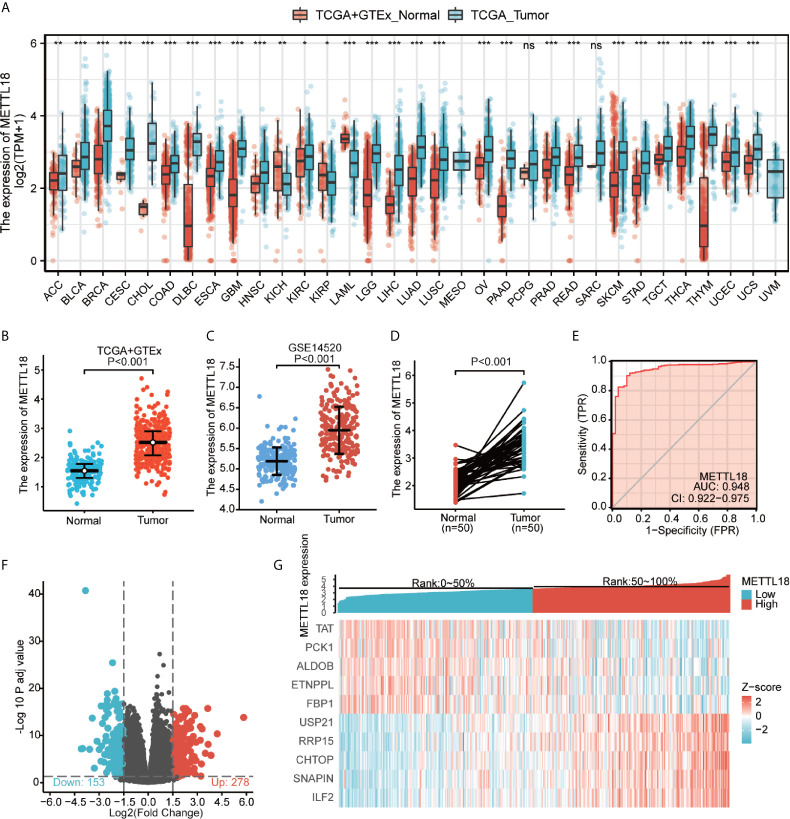
The expression levels of METTL18 in different cancers and METTL18-associated differentially expressed genes (DEGs). **(A)** Increased or decreased METTL18 of different cancers compared with normal tissues in the TCGA and GTEx database. **(B–D)** The expression levels of METTL18 in HCC. **(E)** A ROC curve to test the value of METTL18 to identify HCC tissues was drawn. **(F, G)** Volcano plots of the DEGs and heat map demonstrating the top 10 DEGs. *p < 0.05, **p < 0.01, ***p < 0.001, NS, no significance.

Based on the integrated analysis of TCGA and Genotype-Tissue Expression (GTEx) datasets, we totally included the expression data of 27 tumors in the database and the data were analyzed using the Wilcoxon test. METTL18 was significantly higher expressed in adrenocortical carcinoma (ACC), breast infiltrating carcinoma (BRCA), bladder urothelial carcinoma (BLCA), colon adenocarcinoma (COAD), cholangiocarcinoma (CHOL), cervical squamous cell carcinoma and adenocarcinoma (CESC), diffuse large B cell lymphoma (DLBCL), esophageal carcinoma (ESCA), pleomorphic glioma (GBM), head and neck squamous cell carcinoma (HNSC), renal clear cell carcinoma (KIRC), renal papillary cell carcinoma (KIRP), renal chromophobe cell carcinoma (KICH), brain low grade glioma (LGG), liver hepatocellular carcinoma (LIHC), acute myeloid leukemia (LAML), lung squamous cell carcinoma (LUSC), lung adenocarcinoma (LUAD), ovarian serous cystadenocarcinoma (OV), prostate cancer (PRAD), pancreatic cancer (PAAD), rectum adenocarcinoma (READ), gastric cancer (STAD), skin melanoma (SKCM), thymic cancer (THYM), thyroid cancer (THCA), testicular germ cell tumors (TGCT), uterine sarcoma (UCS), endometrial cancer (UCEC) (P < 0.05; **Figure 1A**). Besides, results have indicated that expression levels of METTL18 in 371 tumor tissues were higher than that in 160 normal samples (P < 0.001; **Figure 1B**) and METTL18 expression in 248 tumor tissues was significantly increased compared with 220 normal samples (P < 0.001; **Figure 1C**). In addition, METTL18 expression values in 50 tumor samples were significantly higher than 50 paired normal samples in the TCGA database *via* analyzing the expression of METTL18 in liver cancer, (P < 0.001; **Figure 1D**). Furthermore, we performed receiver operating characteristic (ROC), the area under the curve (AUC) of METTL18 is 0.948, which indicates that METTL18 was significantly different expression in tumor and normal tissue (**Figure 1E**).

### Identification of DEGs in HCC

We compared 186 HCC METTL18-high samples with 185 METTL18-low samples. Between the two groups, a total of 431 DEGs, including 153 downregulated genes and 278 upregulated genes, were found to be statistically significant (adjusted p-value < 0.05 and absolute Log2-fold change > 1.5) (**Figure 1F** and **Supplementary Table 2**). In addition, applying DESeq2 package, we analyzed DEGs in HTSeq-Counts. Ranked by relative expression, the top 10 differential expressed genes between two groups were demonstrated (**Figure 1G**).

### Enrichment of Biofunction and Analysis of METTL18 Associated Genes in HCC

**Figure 2:**
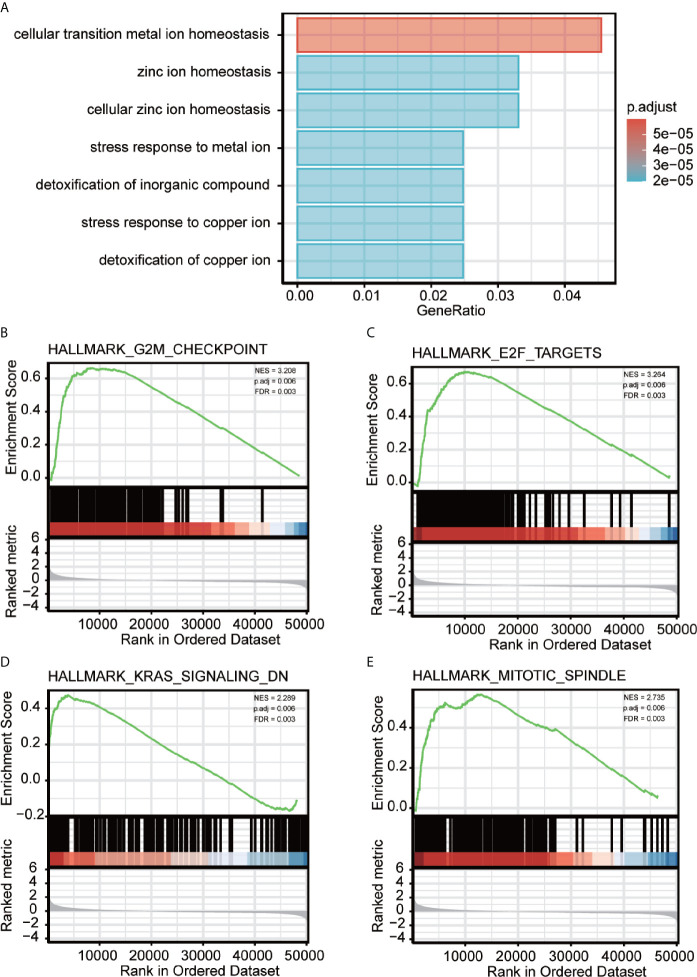
Significantly enriched GO annotations of METTL18 related genes in HCC. **(A)** Top seven of biological process enrichment related to METTL18 related genes with bar graph. **(B–E)** Enrichment plots from the gene set enrichment analysis (GSEA). Several pathways and biological processes were differentially enriched in METTL18-related HCC, including G2M checkpoint, KRAS signaling, mitotic spindle and E2F targets. NES, normalized enrichment score; p. adj, adjusted P value; FDR, false discovery rate.

To better analyze the enrichment of biological function of METTL18 associated genes, we applied Metascape to explore GO enrichment, which demonstrated that METTL18 associated genes were involved in a number of Biological Processes (BPs), Cellular Compositions (CCs), and Molecular Functions (MFs). For instance, different expression of METTL18 could modulate the cellular transition metal ion homeostasis, stress response to metal ion, detoxification of inorganic compound and cellular zinc ion homeostasis. Moreover, cellular response to cadmium ion, kidney development, response to metal ion, appendage morphogenesis, transmembrane receptor protein threonine/serine kinase pathway, and BMP pathway also have the relationship with METTL18 related genes (**Figure 2A** and **Supplementary Table 3**).

### Analysis of Protein–Protein Interaction Network

Using PPI network, we explore the association between the 431 DEGs in HCC group, by the STRING dataset and high confidence (0.70) of interaction score is set. Then, 205 proteins and 322 edges were chosen and with the criteria of total scores ≥5,000, three clusters of hub genes were chosen from PPI network (**Supplementary Figures 1A–D**; **Supplementary Table 4**). In addition, top 10 hub genes included TAT, PCK1, ALDOB, ETNPPL, FBP1, USP21, RRP15, CHTOP, SNAPIN, and ILF2.

### Potential Mechanism of METTL18 in Regulating the Progression of HCC

To explore METTL18-associated pathways in HCC, we applied GSEA between the expression data of METTL18-high and -low to investigate significant differences (nominal, NOM p value < 0.05; false discovery rate, FDR q value < 0.25) in enrichment of the Molecular Signatures Database Collection (MSigDB) (c2.cp.reactome/biocarta/kegg.v6.2.symbols.gmt). We have chosen the top significantly enriched pathways by their normalized enrichment score (NES). Enrichment plots of GSEA revealed that G2M checkpoint, KRAS signaling, mitotic spindle and E2F targets were significantly enriched in patients with METTL18 (**Figures 2B–E**; **Supplementary Table 5**).

### Association Between Expression of METTL18 and Immune Infiltration

**Figure 3:**
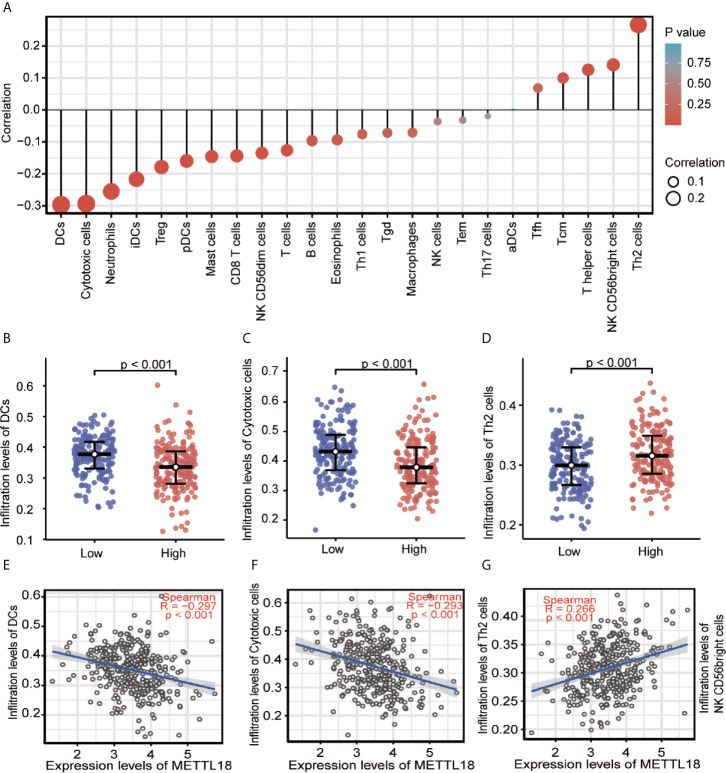
The expression of METTL18 was related to the immune infiltration in the tumor microenvironment. **(A)** Association between the METTL18 expression level and relative abundances of 24 immune cells. The size of dots demonstrates the absolute value of Spearman R. **(B–G)** Correlation diagrams and scatter plots indicating the differentiation of DCs, Cytotoxic cells, and Th2 cells infiltration level between high and low groups of METTL18 expression.

Quantified by ssGSEA in the HCC tumor environment, Spearman correlation has been applied to demonstrate the association between the immune cell infiltration level and the expression level of METTL18. As shown in **Figures 3A–G** (P < 0.05), the expression of METTL18 was positively related to the abundance of acquired immunocytes [Th2 cells (R = 0.266, P < 0.001), T helper cells (R = 0.125, P = 0.016), *etc.*], and negatively associated with the abundance of innate immunocytes [DCs (R = −0.297, P < 0.001), Cytotoxic cells (R = −0.297, P < 0.001), iDCs (R = −0.217, P < 0.001), *etc.*].

### The Connection Between the Expression Level of METTL18 and Clinicopathologic Variables

**Figure 4:**
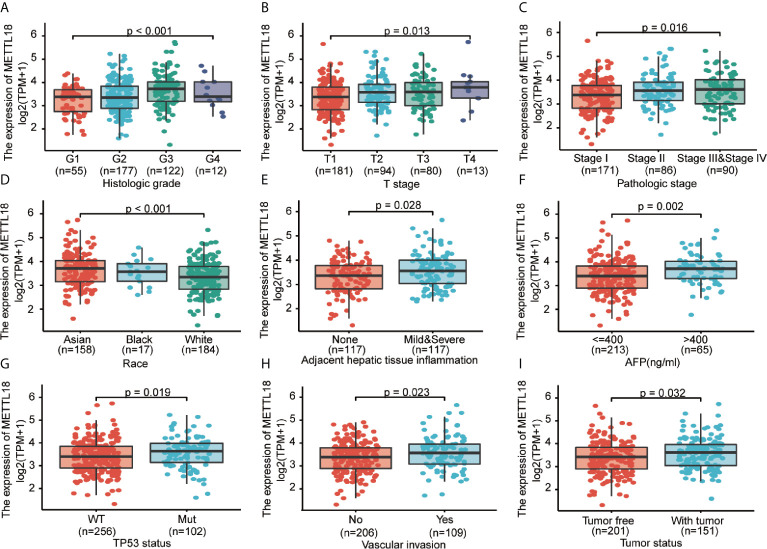
Correlation between METTL18 expression and clinicopathological characteristics, including **(A)** histological grade, **(B)** T stage, **(C)** pathologic stage, **(D)** Race, **(E)** Adjacent hepatic tissue inflammation, **(F)** AFP, **(G)** TP53 status, **(H)** Vascular invasion, and **(I)** Tumor status in HCC patients in TCGA cohort. TCGA, The Cancer Genome Atlas; HCC, hepatocellular cancer.

To explore the significance and role of METTL18 expression, all of 371 HCC patients with patients’ characteristics and METTL18 expression data were identified from TCGA. With an average age of 61.0 years (range from 51.25 to 69.00 years), the cohort containing 250 men and 121 women. As demonstrated in **Table 1** and **Figures 4A–I**, higher expression level of METTL18 was significantly related to T stage (T4 *vs.* T1, P = 0.013), histological grade (grade 4 *vs*. grade 1, P < 0.001), pathologic stage (stages III and IV *vs*. stage I, P = 0.016), Race (White *vs*. Asian, P < 0.001), adjacent hepatic tissue inflammation (Mild and Severe *vs*. None, P = 0.028), AFP (>400 *vs*. <=400, P = 0.002), TP53 status (mutational type *vs*. wild type, P = 0.019), Vascular invasion (yes *vs*. no, P = 0.023), and tumor status (with tumor *vs*. tumor free, P = 0.032). In addition, we performed the univariate logistic regression to explore the association between the expression of METTL18 and prognostic factors (**Table 2**). Higher expression of METTL18 in HCC is positively related to T stage (OR = 1.06 for T2, T3 and T4 *vs*. T1), pathologic stage (OR = 1.05 for Stages II−IV *vs*. Stage I), histological grade (OR = 1.08 for G3 and G4 *vs*. G1 and G2), adjacent hepatic tissue inflammation (OR = 1.06 for Mild and Severe *vs*. None), vascular invasion (OR = 1.05 for yes *vs*. no), race (OR = 1.07 for Asian and Black *vs*. White), and AFP (ng/ml) (OR= 1.04 for >400 *vs*. <=400) (all P < 0.05). Our results demonstrated that HCC patients with higher METTL18 expression were prone to develop to a more advanced stage.

**Table 1:** The correlation between clinicopathological variables and METTL18 expression.

Characters	level	Low expression of METTL18	High expression of METTL18	p	test
n		186	185		
T stage (%)	T1	99(53.8%)	82(44.6%)	0.206	exact
	T2	42(22.8%)	52(28.3%)		
	T3	39(21.2%)	41(22.3%)		
	T4	4(2.2%)	9(4.9%)		
N stage (%)	N0	121(98.4%)	131(98.5%)	1.000	exact
	N1	2(1.6%)	2(1.5%)		
M stage (%)	M0	128(99.2%)	138(97.9%)	0.624	exact
	M1	1(0.8%)	3(2.1%)		
Pathologic stage (%)	Stage I	94(53.7%)	77(44.8%)	0.396	exact
	Stage II	40(22.9%)	46(26.7%)		
	Stage III	39(22.3%)	46(26.7%)		
	Stage IV	2(1.1%)	3(1.7%)		
Residual tumor (%)	R0	167(96.0%)	157(93.5%)	0.390	exact
	R1	7(4.0%)	10(6.0%)		
	R2	0(0.0%)	1(0.6%)		
Histologic grade (%)	G1	33(17.9%)	22(12.1%)	<0.001	
	G2	105(57.1%)	72(39.6%)		
	G3	39(21.2%)	83(45.6%)		
	G4	7(3.8%)	5(2.7%)		
Gender (%)	Female	60(32.3%)	61(33.0%)	0.912	exact
	Male	126(67.7%)	124(67.0%)		
Race (%)	Asian	63(35.4%)	95(52.5%)	0.003	exact
	Black or African American	8(4.5%)	9(5.0%)		
	White	107(60.1%)	77(42.5%)		
Adjacent hepatic tissue inflammation (%)	Mild	44(35.8%)	55(49.5%)	0.076	
	None	70(56.9%)	47(42.3%)		
	Severe	9(7.3%)	9(8.1%)		
Child-Pugh grade (%)	A	113(91.1%)	104(90.4%)	0.907	exact
	B	10(8.1%)	11(9.6%)		
	C	1(0.8%)	0(0.0%)		
Vascular invasion (%)	No	110(68.3%)	96(62.3%)	0.318	
	Yes	51(31.7%)	58(37.7%)		
Tumor status (%)	Tumor free	109(62.3%)	92(52.0%)	0.053	exact
	With tumor	66(37.7%)	85(48.0%)		
TP53 status (%)	Mut	42(23.3%)	60(33.7%)	0.040	
	WT	138(76.7%)	118(66.3%)		
Age (%)	<=60	87(46.8%)	90(48.9%)	0.758	
	>60	99(53.2%)	94(51.1%)		
AFP (ng/ml) (%)	<=400	117(82.4%)	96(70.6%)	0.029	
	>400	25(17.6%)	40(29.4%)		

**Table 2:** METTL18 expression association with clinical pathological characteristics (logistic regression).

Characteristics	the number of patients involved	Odds Ratio (OR)	P value
T stage (T2&T3&T4 vs. T1)	368	1.06(1.02-1.10)	0.002
N stage (N1 vs. N0)	256	0.89(0.68-1.07)	0.326
M stage (M1 vs. M0)	270	1.06(0.93-1.16)	0.283
Pathologic stage (Stage II& Stage III& Stage IV vs. Stage I)	347	1.05(1.02-1.09)	0.005
Histologic grade (G3&G4 vs. G1&G2)	366	1.08(1.04-1.12)	<0.001
Residual tumor (R1&R2 vs. R0)	342	1.03(0.96-1.08)	0.358
Child-Pugh grade (B&C vs. A)	239	0.98(0.90-1.05)	0.654
Adjacent hepatic tissue inflammation (Mild& Severe vs. None)	234	1.06(1.02-1.11)	0.010
Vascular invasion (Yes vs. No)	315	1.05(1.02-1.10)	0.007
Tumor status (With tumor vs. Tumor free)	352	1.03(1.00-1.07)	0.053
TP53 status (Mut vs. WT)	358	1.03(1.00-1.07)	0.062
Gender (Male vs. Female)	371	1.00(0.96-1.03)	0.786
Race (Asian& Black or African American vs. White)	359	1.07(1.04-1.12)	<0.001
Age (>60 vs. <=60)	370	0.98(0.95-1.01)	0.191
AFP (ng/ml) (>400 vs. <=400)	278	1.04(1.00-1.08)	0.027

### Higher Expression of METTL18 Was Significantly Associated With Poor Prognosis of Patients With HCC

**Figure 5:**
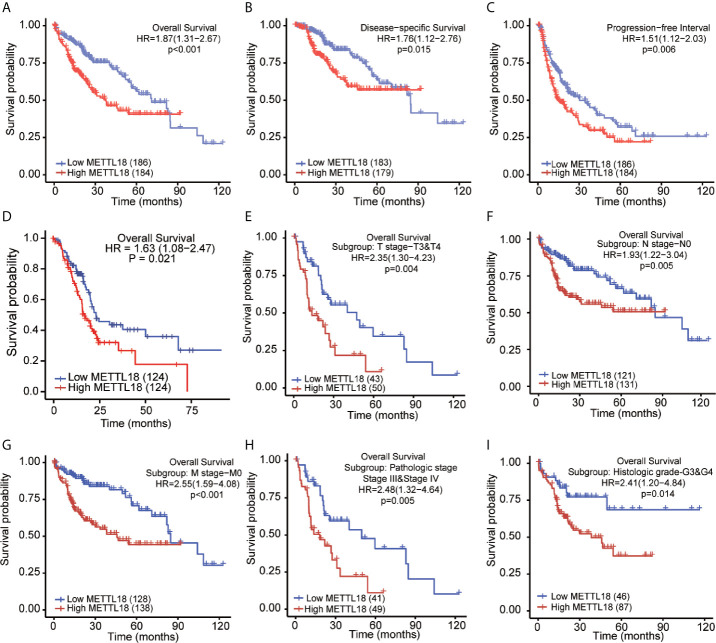
Kaplan–Meier survival plots comparing the low and high expression of METTL18 in HCC. **(A–D)** Survival curves of OS, DSS, and PFI between high and low expression of METTL18 in patients with HCC. **(E–I)** OS survival curves of T stages 3 and 4, N0, M0, pathologic stages III and IV, and G3 and 4 subgroups between METTL18-high and -low patients with HCC. HCC, hepatocellular carcinoma; OS, overall survival; DSS, disease specific survival; PFI, progression free interval.

The OS rates of HCC patients were significantly increased among samples with lower expression of METTL18 than patients with higher expression of METTL18 (P = 0.006; **Figure 5A**). Furthermore, DSS and PFI in lower METTL18 cohort were significantly longer than higher METTL18 cohort (P = 0.015; P = 0.006; **Figures 5B, C**). we also have validated the prognostic value of METTL18 in GEO datasets and the results indicated that higher expression of METTL18 was significantly related to poor prognosis (P = 0.021; **Figure 5D**). Then, based on the information of OS, DSS, and PFI, we performed subgroup analyses of prognosis, which demonstrated that survival rates of HCC samples with higher METTL18 expression was poor in T stages 3–4, N0, M0, and G3−4 subgroups of OS and pathologic stage III−IV subgroup of OS (**Figures 5E–I**). In addition, it should be mentioned that HCC samples with higher METTL18 in pathologic stage III−IV subgroup had worse prognosis in both OS and DSS (P = 0.005; P = 0.044), demonstrating METTL18 as a prognostic factor in HCC samples with distant metastasis. Lamentedly, there was no statistical difference in each subgroup of PFI. Using univariate cox regression, we have demonstrated that increased expression of METTL18 was related to poor OS (HR: 1.870; CI: 1.309−2.671; P = <0.001) (**Table 3**), DSS (HR: 1.76; CI: 1.12−2.76; P = 0.015) (**Supplementary Table 6**), and PFI (HR: 1.51; CI: 1.12−2.03; P = 0.006) (**Supplementary Table 7**). Furthermore, to better explore the characteristics related to prognosis, an analysis of multivariate regression was applied with Tumor status, T stage, M stage, and TP53 status. Similarly, higher expression of METTL18 was still an independent role related to poor OS (HR: 2.055; CI: 1.246−3.389; P = 0.005) (**Table 3**). In addition, increased expression of METTL18 also connected with poor DSS (HR: 2.488; CI: 1.026−6.035; P = 0.044) (**Supplementary Table 6**) while the expression of METTL18 demonstrated no relationship with PFI (HR: 1.114; CI: 0.736−1.687; P = 0.608) (**Supplementary Table 7**) in samples with HCC. According to the results of multivariate Cox regression, we analyzed the influence of METTL18 on prognosis (OS, DSS, and PFI) in subgroups. The higher expression of METTL18 patients demonstrated poor OS in pathologic stage III and IV subgroup (HR: 2.479; CI: 1.323–4.644; P = 0.005), histologic grade 3 and 4 subgroup (HR: 2.407; CI: 1.198–4.837; P = 0.014), T3 and 4 subgroup (HR: 2.348; CI: 1.304–4.227; P = 0.004), N0 subgroup (HR: 1.927; CI: 1.222–3.038; P = 0.005), and M0 subgroup (HR: 2.546; CI: 1.589–4.081; P < 0.001) (**Table 4**). Moreover, the results of subgroup analysis of DSS and PFI also found that higher expression of METTL18 had a worse survival rate in subgroup of other factors (**Supplementary Tables 8**, **9**).

**Table 3:** Univariate and multivariate survival method (Overall Survival) of prognostic covariates in patients with hepatocellular carcinoma.

Characteristics	Total(N)	HR (95% CI) Univariate analysis	P value Univariate analysis	HR (95% CI) Multivariate analysis	P value Multivariate analysis
T stage (T1 vs. T2&T3&T4)	367	0.474(0.330-0.681)	<0.001	0.427(0.264-0.689)	<0.001
N stage (N0 vs. N1)	256	0.499(0.122-2.037)	0.333		
M stage (M0 vs. M1)	270	0.248(0.078-0.789)	0.018	0.368(0.113-1.201)	0.098
Histologic grade (G1&G2 vs. G4&G3)	365	0.893(0.623-1.281)	0.539		
Vascular invasion (No vs. Yes)	314	0.742(0.490-1.124)	0.159		
Residual tumor (R0 vs. R1&R2)	341	0.637(0.322-1.258)	0.194		
Albumin(g/dl) (<3.5 vs. >=3.5)	296	1.085(0.665-1.771)	0.743		
AFP(ng/ml) (<=400 vs. >400)	277	0.947(0.579-1.548)	0.827		
TP53 status (WT vs. Mut)	357	0.697(0.473-1.029)	0.069	0.778(0.478-1.267)	0.313
Child-Pugh grade (A vs. B&C)	238	0.619(0.305-1.254)	0.183		
Race (Asian&Black or African American vs. White)	358	0.803(0.559-1.153)	0.235		
Adjacent hepatic tissue inflammation (None vs. Mild&Severe)	233	0.815(0.501-1.325)	0.409		
Age (<=60 vs. >60)	370	0.802(0.565-1.136)	0.214		
Gender (Female vs. Male)	370	1.225(0.860-1.746)	0.260		
Prothrombin time (<=4 vs. >4)	293	0.752(0.496-1.140)	0.179		
METTL18 (High vs. Low)	370	1.870(1.309-2.671)	<0.001	2.485(1.523-4.053)	<0.001

**Table 4:** The prognostic value of METTL18 (Overall Survival) in diverse hepatocellular carcinoma subgroups.

Characteristics	N (%)	HR (95% CI)	P value
T stage			
T1	181 (49)	1.555(0.869-2.782)	0.137
T2	93 (25)	1.868(0.850-4.105)	0.120
T3&T4	93 (25)	2.348(1.304-4.227)	0.004
N stage			
N0	252 (98)	1.927(1.222-3.038)	0.005
N1	4 (2)	–	–
M stage			
M0	266 (99)	2.546(1.589-4.081)	<0.001
M1	4 (1)	–	–
Pathologic stage			
Stage I	171 (49)	1.556(0.847-2.859)	0.154
Stage II	85 (25)	1.554(0.686-3.520)	0.291
Stage III&Stage IV	90 (26)	2.479(1.323-4.644)	0.005
Histologic grade			
G1	55 (15)	2.089(0.816-5.349)	0.125
G2	177 (48)	1.418(0.842-2.390)	0.189
G4&G3	133 (36)	2.407(1.198-4.837)	0.014

### Construction and Evaluation of a Nomogram Related to METTL18

**Figure 6:**
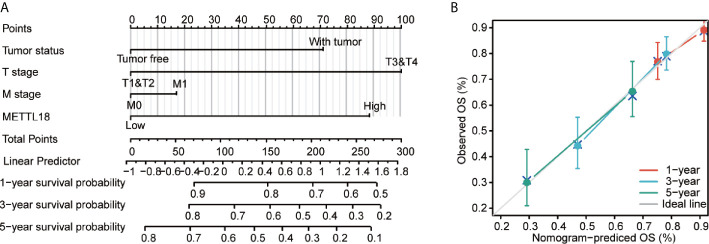
A quantitative method to predict HCC patients’ probability of 1, 3, and 5 year OS. **(A)** A nomogram for estimating the probability of 1, 3, and 5 year OS for HCC patients. **(B)** Calibration plots of the nomogram for evaluating the probability of OS at 1, 3, and 5 years. HCC, hepatocellular carcinoma; OS, overall survival.

To better predict the survival rates of HCC patients, we performed a nomogram based on METTL18 and other independent clinicopathologic factors (**Figure 6A**). The point scale of nomogram was applied to assign points to each variable by the results of multivariate Cox regression. With the adjusted range from 1 to 100, the points of each variable were added up and total scores were calculated. By delineating a direct line down from the total score line to the outcome line, the probable prognosis of each HCC patients at 1-, 3-, and 5-years were defined. For example, an HCC patient with METTL18-high risk (89 points), tumor free (0 points), T3 (100 points), and M0 (0 points) could attain a total point of 189. The survival rates of 1-, 3-, 5-year were about 78.5, 48.5, and 32% (**Figure 6A**). Furthermore, the efficiency of the nomogram has been evaluated, and the calibration curve with Hosmer test of the nomogram in the TCGA-LIHC cohort was 0.689, which indicated that the ability of prediction efficiency of the nomogram is moderately accurate (**Figure 6B**).

### Knockdown of METTL18 Suppress Malignant Phenotype of Hepatocellular Carcinoma *In Vitro*

**Figure 7:**
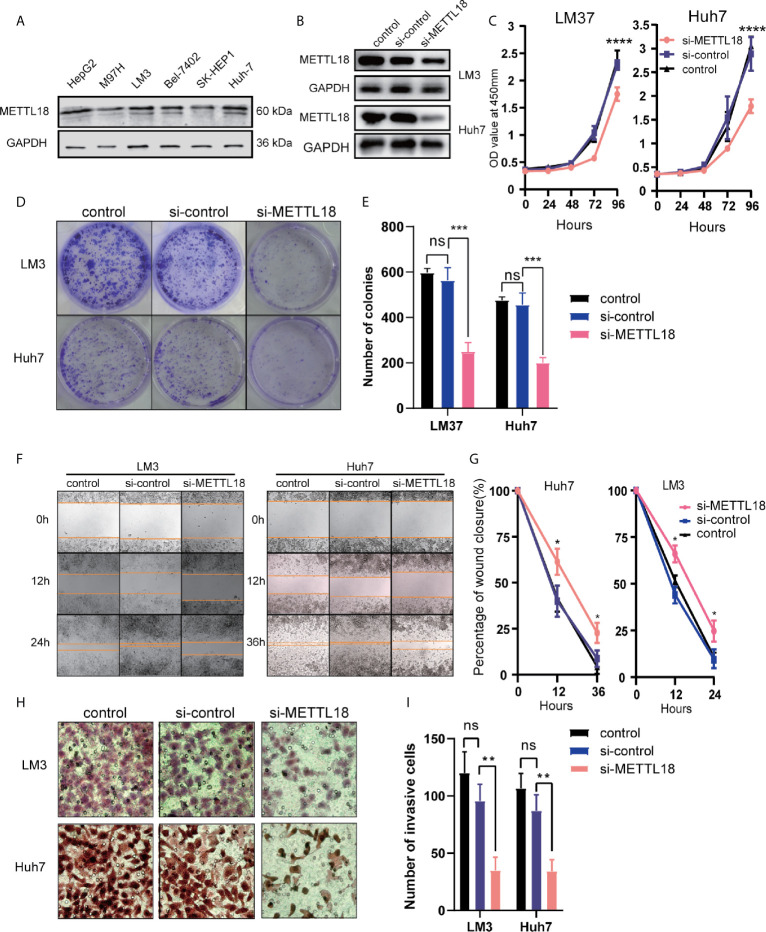
Decreased the expression of METTL18 inhibits proliferation, invasion, and migration of liver cancer cells *in vitro*. **(A)** The expression of METTL18 in the HepG2, M97H, LM3, Bel7402, SK-HEP1, and Huh7 cell lines were detected by western blotting, respectively. **(B)** The transfection efficiency of si-METTL18 in the LM3 and Huh7 cell lines explores by western blotting. **(C)** The CCK-8 assay was applied to detect the efficiency of METTL18 knockdown on the proliferation of LM3 and Huh7 cell lines. **(D)** Images of the colony formation assay after knockdown of METTL18 in the LM3 and Huh7 cell lines. **(E)** Representational statistical analysis of the colony formation assay, including control, si-control, and si-METTL18 groups. **(F)** Representational images of the wound healing assay. **(G)** Statistical analysis of the wound healing assay results after decreased expression of METTL18. **(H)** Images of the transwell assay results after knockdown of METTL18 in the LM3 and Huh7 cell lines. **(I)** Representational statistical analysis of the transwell assay. *p < 0.05, **p < 0.01, ***p < 0.001, ****p < 0.0001, NS, no significance.

To explore the role of METTL18 in LIHC, the expression of METTL18 in the six hepatocellular carcinoma cell lines (HepG2, M97H, LM3, Bel7402, SK-HEP1, and Huh7) was analyzed respectively. The expression of METTL18 in the LM3 and Huh7 cell lines was higher than other cell lines (**Figure 7A**). Then, the LM3 and Huh7 cell lines were selected for functional analysis. Using western blotting, we have detected the efficiency of METTL18-siRNA (**Figure 7B**). The results of colony formation and CCK8 experiments demonstrated that lower expression of METTL18 significantly inhibited the ability of proliferation and colony formation of LM3 and Huh7 cells (**Figures 7C–E**). The results of wound healing experiment indicated that knockdown of METTL18 significantly inhibited the migration of LM3 and Huh7 cells (**Figures 7F, G**). The results of transwell experiment indicated that the LM3 and Huh7 cells exhibited significantly decreased invasion ability upon METTL18 knockdown (**Figures 7H, I**). These results proved that METTL18 could be a promoter of hepatocellular carcinoma. Further research studies are needed to reveal the underlying mechanisms.

## Discussion

More than 27 members are involved in the METTL family, but only a few of them have been studied (15, 24–26). Since the METTL family shares a limited conserved domain, it is found that their functions are diverse (24). Recently research studies have demonstrated that members of METTL play a key role in the progression of a number of tumors *via* multiple mechanisms. For example, METTL3 could induce m6A modification in coding region of mRNA transcription related to the cell cycle, which is essential for the differentiation of leukemia cells and ultimately promotes the translation of a variety of oncogenic mRNAs (27). In addition, a number of members of METTL family have been demonstrated participating in the process of initiation and development of cancers (11–13). For instance, METTL16, METTL2B, and METTL8 (14, 15) and play significant roles in tumorigenesis (16). However, the impact of METTL18 in tumor development has not been explored.

Based on our study, the expression levels and prognostic values of METTL18 were evaluated, we found that expression of METTL18 is abnormal in a number of tumors and significantly high in liver cancer in multiple databases. In addition, we found that METTL18 has a relatively high ROC score with an AUC of 0.948 for HCC in the TCGA database. In general, METTL18 is differentially expressed in tumor and normal samples. However, further prospective research studies are warranted to form the diagnostic accuracy of METTL18 in HCC.

To better explore the METTL18 function, we used GO and GSEA to perform functional analysis. We found that higher METTL18 phenotype was associated with cellular zinc ion homeostasis, detoxification of copper ion, stress response to copper ion, zinc ion homeostasis by GO analysis. Recently, multiple studies have demonstrated that alterations of metal molecular, such as Zinc and Copper, could modulate various molecular targets which playing an important role in progression and development in various cancers, such as cyclic AMP (cAMP)-dependent protein kinase (PKA), protein kinase C (PKC), and transcription factors (including nuclear factor (NF)-*κ*B) (28–32). In addition, using Cytoscape, the PPI network of METTL18-related genes was performed, and the results indicated that diverse biological processes and signaling pathways have association with these genes. In future research studies, we will explore the relationship between METTL18-related genes and prognosis of HCC.

We also indicated that METTL18 was significantly associated with G2M checkpoint, E2F targets, KRAS signaling, and mitotic spindle by GSEA. G2M checkpoint and KRAS signaling pathway have been proved to play an important role in the progression and development of liver cancer (33–35). A previous study has demonstrated biological processes related to cell proliferation like G2M checkpoint, and cell cycle were enriched in abnormal expression of RNA-methyltransferase NSUN6 (36). Furthermore, research studies have demonstrated that E2Fs are the downstream of cell cycle signaling pathway and have a significant impact in modulation of cell proliferation, differentiation, and apoptosis (37–39). Also, mitotic spindle has been found enhancing chromosomal instability and liver cancer progression (35). These studies and our results indicated that METTL18 might contribute to HCC initiation and development by modulating cell cycle and KRAS pathway. But the association of METTL18 expression with G2M checkpoint, KRAS signaling, mitotic spindle and E2F targets was the first to be reported, and the regulatory molecular mechanism needs to be further explored.

Recently, research studies have shown that tumor infiltrating immune cells (TIICs) could modulate the process of development as well as progression of tumor (40). In addition, TIICs are potentially clonally expanded and preferentially enriched in HCC (41), and poor prognosis correlates with accumulation of TIICs in HCC (42). Then, our results demonstrated that METTL18 expression in HCC was negatively associated with multiple types of immune cell infiltration, for example, dendritic cells (DCs, iDCs, and pDCs) and cytotoxic cells. DCs, also known as a group of antigen-presenting cells, have a significant impact on the initiation and regulation of cancer immune responses (43). Recently, one study has shown DCs can generate resistance to HCC (44). Immature DCs have the phagocytic ability. However, mature DCs have a significant modulation function as well as produce a large number of cytokines (45). These results demonstrated that the DCs had a potent negatively correlation with METTL18. In addition, cytotoxic cells, also known as CD8^+^ T lymphocytes with cytotoxic granules, are the important anti-tumor effector cells (46). One research has reported that hepatocellular carcinoma cells could mediate the progression, development and tumor resistance to PD1 by inhibiting the cytotoxic T cell response (47). Therefore, our results reveal the potent modulating role of METTL18 in immune response with liver cancer. Furthermore, significantly connection can be found between expression of METTL18 and the modulation of T helper cells, Th2 cells, and Tfh in HCC. These associations could be indicative of a potent mechanism where METTL18 modulates T cell functions in HCC. In summary, these results demonstrate that the METTL18 plays a significant role in modulation of immune infiltrating cells in HCC.

Although research studies have demonstrated that there was diverse association between the expression of member of METTL and clinical pathological factors in HCC (17, 18, 48). In our study, results have indicated that elevated expression level of METTL18 was related to poor prognosis and advanced clinical pathologic characteristics in HCC. In a stratified analysis, we found that METTL18 expression remained a powerful factor to forecast the prognosis within these subgroups, such as T2 to 4, stage 2 to 4, N0, M0 and histologic grade G3 and G4 *etc*, indicating that METTL18 was independent of these significant clinicopathological parameters.

After regulating the conventional clinicopathological factors, our study indicates that METTL18 could perform as an independent prognostic factor of poor OS and DSS for HCC. Then, our METTL18 related nomogram was performed with the expression level of METTL18 and other clinical factors (cancer status, T stage, M stage, *etc*). The C-index of METTL18-related Cox model for overall survival prediction was 0.689 (95%CI: 0.645−0.732). The calibration plots demonstrated optimal agreement between the prediction by METTL18-related nomogram and actual observation for 1-, 3-, and 5-year OS probability. Our nomogram was performed based on the complementary perspective for individual patients and provided a personalized score for respective tumors. In addition, our model could be a new method to estimate the prognosis of clinicians in the future.

Furthermore, the impact of METTL18 on the ability of proliferation, invasion, and migration of hepatocellular carcinoma cells was explored *in vitro*. We found that malignant phenotype of HCC cells was suppressed when METTL18 is knocked down, indicating the expression of METTL18 is a potent target for hepatocellular cancer therapy. In future research studies, the underlying mechanisms of METTL18 in HCC should be elucidated.

Although this research improved our understanding about the correlation between METTL18 and HCC, there still existed some limitations. Firstly, to better evaluate the significant role of METTL18 in progression of hepatocellular carcinoma, all types of clinical characteristics should be involved, for example, the way of treatment for each patient. However, because the experiments were done in different center, this kind of missing information were inevitable in public databases. Second, the relationship between the expression level of METTL18 and prognosis should be verified using more clinical samples, and using the single cohort from public datasets to predict the prognosis is far from perfect. In addition, because the limitations of this research design, the significant pathways correlated with METTL18 may have been missed, and the related signaling should be explored further. To better examine the mechanism of METTL18 in HCC, we would conduct more experimental studies on METTL18 in the sooner future.

## Conclusion

In our study, we demonstrated METTL18 as an important molecular biomarker with prognostic value and may have significant impact on the modulation of cell cycle, KRAS signaling and immune infiltration in HCC. This research supported promising visions for subsequent study to clarify the molecular pathogenesis and clinicopathological significance of HCC. Randomized clinical trials and further studies are needed to analyze the underlying mechanism and clinical applications for HCC patients.

## Data Availability Statement

The original contributions presented in the study are included in the article/**Supplementary Material**. Further inquiries can be directed to the corresponding author.

## Ethics Statement

The ethics consent and approval to participate in the current study was approved by the ethics committee of Peking Union Medical College Hospital.

## Author Contributions

T-HL and CQ contributed equally to this article. T-HL designed the research and participated in manuscript writing. B-BZ, CQ, H-TC, and X-TZ participated in generating and analyzing the information. X-YY, Y-YW, and Z-RL assisted with analysis. All authors contributed to the article and approved the submitted version.

## Conflict of Interest

The authors declare that the research was conducted in the absence of any commercial or financial relationships that could be construed as a potential conflict of interest.

## Supplementary Material

Supplementary Figure 1 The interactions network of protein to protein built based on interactions pairs of protein to protein by the STRING dataset.

Supplementary Table 1 TCGA HCC patient characteristics.

Supplementary Table 2 METTL18-related differential expressed Genes.

Supplementary Table 3 METTL18 of Gene ontology (GO) enrichment analysis.

Supplementary Table 4 The METTL18 protein to protein interactions.

Supplementary Table 5 METTL18-Gene sets enriched in phenotype high. p. adjusted, adjusted P-value; NES, normalized enrichment score.

Supplementary Table 6 Univariate and multivariate survival results (Disease Specific Survival) of prognostic covariates in HCC patients.

Supplementary Table 7 Univariate and multivariate survival results (Progression Free Interval) of prognostic covariates in HCC patients.

Supplementary Table 8 The prognostic value of METTL18 (Disease Specific Survival) in various HCC subgroups.

Supplementary Table 9 The prognostic value of METTL18 (Progression Free Interva) in various HCC subgroups.
