# Supplementary figures and images for: Identification METTL18 as a Potential Prognosis Biomarker and Associated With Immune Infiltrates in Hepatocellular Carcinoma
Source: Front Oncol. 2021 May 26;11:665192. doi: 10.3389/fonc.2021.665192 (PMC8187872; doi:10.3389/fonc.2021.665192)

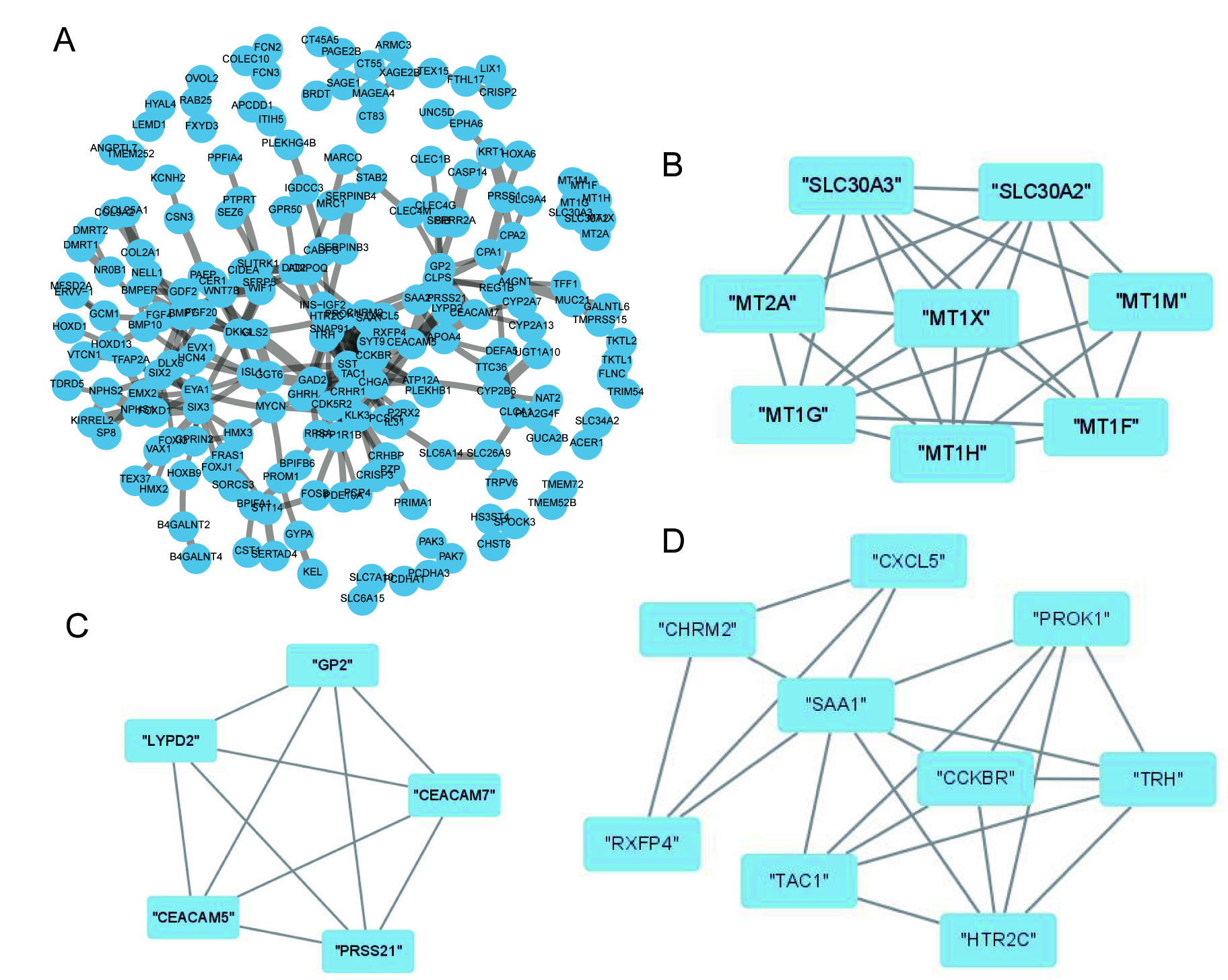

Supplement: Supplementary Figure 1 — The interactions network of protein to protein built based on interactions pairs of protein to protein by the STRING dataset. [file Image_1.tif]
