# Supplementary material for: Identification METTL18 as a Potential Prognosis Biomarker and Associated With Immune Infiltrates in Hepatocellular Carcinoma
Source: Front Oncol. 2021 May 26;11:665192. doi: 10.3389/fonc.2021.665192 (PMC8187872; doi:10.3389/fonc.2021.665192)
Supplement: Supplementary Table 1 — TCGA HCC patient characteristics. [file Table_1.docx]

| Characters | level | Overall |
| --- | --- | --- |
| n |  | 371 |
| T stage (%) | T1 | 181(49.2%) |
|  | T2 | 94(25.5%) |
|  | T3 | 80(21.7%) |
|  | T4 | 13(3.5%) |
| N stage (%) | N0 | 252(98.4%) |
|  | N1 | 4(1.6%) |
| M stage (%) | M0 | 266(98.5%) |
|  | M1 | 4(1.5%) |
| Pathologic stage (%) | Stage I | 171(49.3%) |
|  | Stage II | 86(24.8%) |
|  | Stage III | 85(24.5%) |
|  | Stage IV | 5(1.4%) |
| Residual tumor (%) | R0 | 324(94.7%) |
|  | R1 | 17(5.0%) |
|  | R2 | 1(0.3%) |
| Histologic grade (%) | G1 | 55(15.0%) |
|  | G2 | 177(48.4%) |
|  | G3 | 122(33.3%) |
|  | G4 | 12(3.3%) |
| Gender (%) | Female | 121(32.6%) |
|  | Male | 250(67.4%) |
| Race (%) | Asian | 158(44.0%) |
|  | Black or African American | 17(4.7%) |
|  | White | 184(51.3%) |
| Adjacent hepatic tissue inflammation (%) | Mild | 99(42.3%) |
|  | None | 117(50.0%) |
|  | Severe | 18(7.7%) |
| Child-Pugh grade (%) | A | 217(90.8%) |
|  | B | 21(8.8%) |
|  | C | 1(0.4%) |
| Vascular invasion (%) | No | 206(65.4%) |
|  | Yes | 109(34.6%) |
| Tumor status (%) | Tumor free | 201(57.1%) |
|  | With tumor | 151(42.9%) |
| TP53 status (%) | Mut | 102(28.5%) |
|  | WT | 256(71.5%) |
| Age (%) | <=60 | 177(47.8%) |
|  | >60 | 193(52.2%) |
| AFP(ng/ml) (%) | <=400 | 213(76.6%) |
|  | >400 | 65(23.4%) |
