# Supplementary material for: Identification METTL18 as a Potential Prognosis Biomarker and Associated With Immune Infiltrates in Hepatocellular Carcinoma
Source: Front Oncol. 2021 May 26;11:665192. doi: 10.3389/fonc.2021.665192 (PMC8187872; doi:10.3389/fonc.2021.665192)
Supplement: Supplementary Table 2 — METTL18-related differential expressed Genes. [file Table_2.docx]

| gene_id | baseMean | log2FoldChange | lfcSE | stat | pvalue | padj | gene_name | gene_biotype | cor_pvalue | correlation |
| --- | --- | --- | --- | --- | --- | --- | --- | --- | --- | --- |
| ENSG00000163631 | 2491759.8 | -0.73962178 | 0.13890177 | -5.32478288 | 1.010737e-07 | 2.196039e-06 | ALB | protein_coding | 2.051987e-04 | -0.19193325 |
| ENSG00000197249 | 983453.0 | -0.46575286 | 0.11330307 | -4.11068181 | 3.944925e-05 | 3.550732e-04 | SERPINA1 | protein_coding | 4.667125e-01 | -0.03788672 |
| ENSG00000198804 | 800718.8 | -0.69677798 | 0.08712991 | -7.99700054 | 1.274867e-15 | 3.600892e-13 | MT-CO1 | protein_coding | 5.778263e-08 | -0.27821506 |
| ENSG00000198886 | 609262.0 | -0.63760279 | 0.08723766 | -7.30880178 | 2.695350e-13 | 3.539661e-11 | MT-ND4 | protein_coding | 7.538667e-07 | -0.25433347 |
| ENSG00000158874 | 568743.5 | -0.32489257 | 0.16358520 | -1.98607562 | 4.702493e-02 | 1.201349e-01 | APOA2 | protein_coding | 7.171414e-01 | 0.01886205 |
| ENSG00000087086 | 546214.6 | -0.20766716 | 0.11802244 | -1.75955665 | 7.848301e-02 | 1.779405e-01 | FTL | protein_coding | 5.739617e-02 | 0.09875992 |
| ENSG00000125730 | 517417.6 | -0.53896178 | 0.10355877 | -5.20440478 | 1.946194e-07 | 3.839417e-06 | C3 | protein_coding | 1.571443e-02 | -0.12539168 |
| ENSG00000171560 | 509105.5 | -0.85929256 | 0.13749804 | -6.24948958 | 4.117962e-10 | 1.998757e-08 | FGA | protein_coding | 4.652061e-11 | -0.33496288 |
| ENSG00000198938 | 440273.4 | -0.62674767 | 0.08490410 | -7.38183025 | 1.561282e-13 | 2.231504e-11 | MT-CO3 | protein_coding | 6.352230e-07 | -0.25599842 |
| ENSG00000210082 | 440267.5 | -0.86409219 | 0.10091401 | -8.56265811 | 1.102946e-17 | 5.377090e-15 | MT-RNR2 | Mt_rRNA | 6.530234e-05 | -0.20619100 |
| ENSG00000257017 | 390337.7 | -1.10010497 | 0.18306563 | -6.00934743 | 1.862716e-09 | 7.182252e-08 | HP | protein_coding | 9.766564e-13 | -0.36120737 |
| ENSG00000198712 | 377277.5 | -0.68391870 | 0.08459807 | -8.08432932 | 6.250722e-16 | 1.951377e-13 | MT-CO2 | protein_coding | 8.023076e-09 | -0.29517833 |
| ENSG00000130203 | 364024.4 | -0.62574322 | 0.11088617 | -5.64311352 | 1.670022e-08 | 4.690955e-07 | APOE | protein_coding | 6.116399e-03 | -0.14219116 |
| ENSG00000198727 | 355911.5 | -0.71750526 | 0.09057987 | -7.92124413 | 2.351457e-15 | 6.198964e-13 | MT-CYB | protein_coding | 1.201966e-09 | -0.31053187 |
| ENSG00000118137 | 351010.1 | -0.89978550 | 0.17293396 | -5.20305831 | 1.960353e-07 | 3.865208e-06 | APOA1 | protein_coding | 8.695951e-06 | -0.22920498 |
| ENSG00000091513 | 344899.6 | -0.65368013 | 0.13593494 | -4.80877183 | 1.518604e-06 | 2.239768e-05 | TF | protein_coding | 1.698998e-02 | -0.12391801 |
| ENSG00000171564 | 324301.6 | -0.92231696 | 0.14819280 | -6.22376368 | 4.853680e-10 | 2.293992e-08 | FGB | protein_coding | 1.037402e-10 | -0.32914106 |
| ENSG00000171557 | 285261.1 | -0.76567222 | 0.14317241 | -5.34790341 | 8.897893e-08 | 1.966876e-06 | FGG | protein_coding | 1.210043e-07 | -0.27157004 |
| ENSG00000106927 | 283379.7 | -0.66341153 | 0.12046016 | -5.50731084 | 3.643566e-08 | 9.131751e-07 | AMBP | protein_coding | 7.341863e-04 | -0.17477564 |
| ENSG00000109072 | 276958.3 | -0.45689689 | 0.11336641 | -4.03026692 | 5.571355e-05 | 4.741247e-04 | VTN | protein_coding | 7.936654e-02 | -0.09120194 |
| ENSG00000198763 | 254605.6 | -0.55134264 | 0.09477391 | -5.81745190 | 5.975145e-09 | 1.954498e-07 | MT-ND2 | protein_coding | 6.565123e-05 | -0.20612685 |
| ENSG00000138207 | 242320.5 | -0.88801067 | 0.12653852 | -7.01771026 | 2.255336e-12 | 2.201171e-10 | RBP4 | protein_coding | 1.718415e-13 | -0.37194040 |
| ENSG00000198899 | 234423.5 | -0.67261363 | 0.08743794 | -7.69246885 | 1.443226e-14 | 2.951895e-12 | MT-ATP6 | protein_coding | 6.274892e-09 | -0.29721599 |
| ENSG00000198888 | 227244.8 | -0.54121066 | 0.08913104 | -6.07207854 | 1.262651e-09 | 5.231258e-08 | MT-ND1 | protein_coding | 2.927270e-03 | -0.15421103 |
| ENSG00000130208 | 221019.8 | -0.68582692 | 0.13413853 | -5.11282562 | 3.173753e-07 | 5.837244e-06 | APOC1 | protein_coding | 5.413091e-04 | -0.17901687 |
| ENSG00000084674 | 218016.6 | -0.38972068 | 0.10887901 | -3.57939232 | 3.443941e-04 | 2.182839e-03 | APOB | protein_coding | 5.642053e-01 | -0.03001760 |
| ENSG00000091583 | 199801.8 | -0.58798503 | 0.13767628 | -4.27077938 | 1.947910e-05 | 1.958310e-04 | APOH | protein_coding | 2.119817e-03 | -0.15922305 |
| ENSG00000120885 | 196253.1 | -0.48637381 | 0.10352114 | -4.69830427 | 2.623304e-06 | 3.544449e-05 | CLU | protein_coding | 4.789872e-01 | -0.03685108 |
| ENSG00000115414 | 191255.5 | -0.04718779 | 0.08722103 | -0.54101386 | 5.884980e-01 | 7.331300e-01 | FN1 | protein_coding | 8.323418e-04 | 0.17300164 |
| ENSG00000110245 | 188077.5 | -0.95862204 | 0.16079843 | -5.96163812 | 2.497217e-09 | 9.226317e-08 | APOC3 | protein_coding | 2.577411e-10 | -0.32237667 |
| ENSG00000136872 | 180737.1 | -1.08641553 | 0.17982791 | -6.04141785 | 1.527658e-09 | 6.126412e-08 | ALDOB | protein_coding | 0.000000e+00 | -0.41053845 |
| ENSG00000135821 | 161973.3 | 0.28121187 | 0.20703246 | 1.35829844 | 1.743690e-01 | 3.219791e-01 | GLUL | protein_coding | 1.923343e-12 | 0.35682632 |
| ENSG00000198786 | 147841.7 | -0.86087262 | 0.11402073 | -7.55014102 | 4.347872e-14 | 7.264621e-12 | MT-ND5 | protein_coding | 3.729146e-07 | -0.26110655 |
| ENSG00000145192 | 146523.9 | -0.28584322 | 0.17172780 | -1.66451338 | 9.600988e-02 | 2.075248e-01 | AHSG | protein_coding | 4.069080e-01 | -0.04316710 |
| ENSG00000117601 | 138731.8 | -0.43164819 | 0.16809289 | -2.56791465 | 1.023123e-02 | 3.600864e-02 | SERPINC1 | protein_coding | 6.151050e-02 | -0.09718191 |
| ENSG00000130649 | 134904.9 | -0.41834488 | 0.26396727 | -1.58483619 | 1.130036e-01 | 2.345136e-01 | CYP2E1 | protein_coding | 3.802842e-01 | -0.04566489 |
| ENSG00000143819 | 134211.3 | -0.18377390 | 0.13238719 | -1.38815468 | 1.650900e-01 | 3.096546e-01 | EPHX1 | protein_coding | 6.444354e-02 | 0.09610939 |
| ENSG00000175899 | 133079.2 | -0.24841448 | 0.15466761 | -1.60611830 | 1.082479e-01 | 2.271081e-01 | A2M | protein_coding | 4.318499e-01 | 0.04091395 |
| ENSG00000135744 | 132483.6 | -0.24512988 | 0.09581874 | -2.55826666 | 1.051954e-02 | 3.685204e-02 | AGT | protein_coding | 1.045642e-01 | 0.08439783 |
| ENSG00000075624 | 126666.8 | -0.00508431 | 0.05600606 | -0.09078142 | 9.276663e-01 | 9.598412e-01 | ACTB | protein_coding | 4.140809e-14 | 0.38007694 |
| ENSG00000145321 | 120845.4 | -0.47042283 | 0.11303045 | -4.16191220 | 3.155937e-05 | 2.940057e-04 | GC | protein_coding | 7.452218e-02 | -0.09270569 |
| ENSG00000229314 | 120232.2 | -0.75204051 | 0.15167447 | -4.95825382 | 7.112959e-07 | 1.170875e-05 | ORM1 | protein_coding | 2.091381e-04 | -0.19168791 |
| ENSG00000167244 | 119560.4 | 0.67998160 | 0.31480806 | 2.15998792 | 3.077360e-02 | 8.627712e-02 | IGF2 | protein_coding | 6.436395e-01 | 0.02408486 |
| ENSG00000156508 | 119363.8 | 0.06024303 | 0.06862681 | 0.87783516 | 3.800332e-01 | 5.519507e-01 | EEF1A1 | protein_coding | 2.594157e-11 | 0.33912210 |
| ENSG00000113889 | 116323.5 | -0.61369506 | 0.14187629 | -4.32556464 | 1.521416e-05 | 1.584597e-04 | KNG1 | protein_coding | 1.102179e-04 | -0.19979391 |
| ENSG00000149131 | 112985.1 | -0.71817050 | 0.10129257 | -7.09006097 | 1.340529e-12 | 1.416178e-10 | SERPING1 | protein_coding | 3.876023e-08 | -0.28173508 |
| ENSG00000110169 | 111541.5 | -1.07509419 | 0.14932754 | -7.19957095 | 6.040232e-13 | 7.048060e-11 | HPX | protein_coding | 1.736501e-13 | -0.37187812 |
| ENSG00000118271 | 107379.3 | -0.89297925 | 0.17642635 | -5.06148456 | 4.160044e-07 | 7.346316e-06 | TTR | protein_coding | 1.309193e-08 | -0.29107152 |
| ENSG00000113905 | 107076.4 | -0.76967309 | 0.20116699 | -3.82604059 | 1.302208e-04 | 9.687352e-04 | HRG | protein_coding | 1.852219e-06 | -0.24540169 |
| ENSG00000166710 | 105152.4 | -0.40822292 | 0.08838990 | -4.61843385 | 3.866472e-06 | 4.923216e-05 | B2M | protein_coding | 3.533003e-01 | 0.04830837 |
