# Supplementary material for: Identification METTL18 as a Potential Prognosis Biomarker and Associated With Immune Infiltrates in Hepatocellular Carcinoma
Source: Front Oncol. 2021 May 26;11:665192. doi: 10.3389/fonc.2021.665192 (PMC8187872; doi:10.3389/fonc.2021.665192)
Supplement: Supplementary Table 3 — METTL18 of Gene ontology (GO) enrichment analysis. [file Table_3.docx]

| ONTOLOGY | ID | Description | GeneRatio | BgRatio | pvalue | p.adjust | qvalue | geneID | Count |
| --- | --- | --- | --- | --- | --- | --- | --- | --- | --- |
| BP | GO:0006882 | cellular zinc ion homeostasis | 8/242 | 36/18670 | 1.570359e-08 | 1.730394e-05 | 1.573294e-05 | MT1H/MT1G/MT1X/MT1M/MT2A/MT1F/SLC30A2/SLC30A3 | 8 |
| BP | GO:0010273 | detoxification of copper ion | 6/242 | 15/18670 | 2.023443e-08 | 1.730394e-05 | 1.573294e-05 | MT1H/MT1G/MT1X/MT1M/MT2A/MT1F | 6 |
| BP | GO:1990169 | stress response to copper ion | 6/242 | 15/18670 | 2.023443e-08 | 1.730394e-05 | 1.573294e-05 | MT1H/MT1G/MT1X/MT1M/MT2A/MT1F | 6 |
| BP | GO:0055069 | zinc ion homeostasis | 8/242 | 38/18670 | 2.481742e-08 | 1.730394e-05 | 1.573294e-05 | MT1H/MT1G/MT1X/MT1M/MT2A/MT1F/SLC30A2/SLC30A3 | 8 |
| BP | GO:0061687 | detoxification of inorganic compound | 6/242 | 17/18670 | 4.895741e-08 | 2.275703e-05 | 2.069095e-05 | MT1H/MT1G/MT1X/MT1M/MT2A/MT1F | 6 |
| BP | GO:0097501 | stress response to metal ion | 6/242 | 17/18670 | 4.895741e-08 | 2.275703e-05 | 2.069095e-05 | MT1H/MT1G/MT1X/MT1M/MT2A/MT1F | 6 |
| BP | GO:0046916 | cellular transition metal ion homeostasis | 11/242 | 108/18670 | 1.578766e-07 | 6.290255e-05 | 5.719169e-05 | GDF2/MT1H/MT1G/SLC22A17/MT1X/MT1M/MT2A/MT1F/SLC30A2/SLC30A3/FTHL17 | 11 |
| BP | GO:0072073 | kidney epithelium development | 12/242 | 140/18670 | 2.888173e-07 | 1.006889e-04 | 9.154750e-05 | ADIPOQ/BMPER/BMP7/WNT7B/FOXD1/FOXJ1/EYA1/NPHS2/NPHS1/CER1/SIX2/PROM1 | 12 |
| BP | GO:0071294 | cellular response to zinc ion | 6/242 | 23/18670 | 3.741162e-07 | 1.060983e-04 | 9.646576e-05 | MT1H/MT1G/MT1X/MT1M/MT2A/MT1F | 6 |
| BP | GO:0010043 | response to zinc ion | 8/242 | 53/18670 | 3.804171e-07 | 1.060983e-04 | 9.646576e-05 | MT1H/MT1G/MT1X/MT1M/MT2A/MT1F/SLC30A2/SLC30A3 | 8 |
| BP | GO:0003002 | regionalization | 18/242 | 351/18670 | 8.093100e-07 | 2.051969e-04 | 1.865672e-04 | EMX2/WNT7B/DKK1/FOXD1/HOXA6/EVX1/HOXD13/ISL1/FOXJ1/CER1/SIX2/SIX3/OVOL2/HOXB9/TDRD5/DMRT2/ALX1/SP8 | 18 |
| BP | GO:0055076 | transition metal ion homeostasis | 11/242 | 131/18670 | 1.117912e-06 | 2.598214e-04 | 2.362325e-04 | GDF2/MT1H/MT1G/SLC22A17/MT1X/MT1M/MT2A/MT1F/SLC30A2/SLC30A3/FTHL17 | 11 |
| BP | GO:0007389 | pattern specification process | 20/242 | 446/18670 | 1.590538e-06 | 3.285890e-04 | 2.987567e-04 | EMX2/BMP7/WNT7B/DKK1/FOXD1/HOXA6/EVX1/HOXD13/ISL1/FOXJ1/EYA1/CER1/SIX2/SIX3/OVOL2/HOXB9/TDRD5/DMRT2/ALX1/SP8 | 20 |
| BP | GO:0071280 | cellular response to copper ion | 6/242 | 29/18670 | 1.649425e-06 | 3.285890e-04 | 2.987567e-04 | MT1H/MT1G/MT1X/MT1M/MT2A/MT1F | 6 |
| BP | GO:0001656 | metanephros development | 9/242 | 90/18670 | 2.523899e-06 | 4.568505e-04 | 4.153735e-04 | ADIPOQ/BMP7/WNT7B/FOXD1/FOXJ1/EYA1/NPHS2/FRAS1/SIX2 | 9 |
| BP | GO:0072311 | glomerular epithelial cell differentiation | 5/242 | 18/18670 | 2.620870e-06 | 4.568505e-04 | 4.153735e-04 | ADIPOQ/FOXJ1/NPHS2/NPHS1/PROM1 | 5 |
| BP | GO:0048706 | embryonic skeletal system development | 10/242 | 126/18670 | 5.751936e-06 | 9.342801e-04 | 8.494577e-04 | BMP7/HOXD1/HOXA6/TFAP2A/COL2A1/EYA1/SIX2/HOXB9/DMRT2/ALX1 | 10 |
| BP | GO:0072010 | glomerular epithelium development | 5/242 | 21/18670 | 6.029775e-06 | 9.342801e-04 | 8.494577e-04 | ADIPOQ/FOXJ1/NPHS2/NPHS1/PROM1 | 5 |
| BP | GO:0001655 | urogenital system development | 16/242 | 330/18670 | 6.848921e-06 | 9.383338e-04 | 8.531434e-04 | ADIPOQ/EMX2/BMPER/BMP7/WNT7B/FOXD1/HOXD13/TFAP2A/FOXJ1/EYA1/NPHS2/NPHS1/CER1/FRAS1/SIX2/PROM1 | 16 |
| BP | GO:0072001 | renal system development | 15/242 | 293/18670 | 6.974019e-06 | 9.383338e-04 | 8.531434e-04 | ADIPOQ/EMX2/BMPER/BMP7/WNT7B/FOXD1/TFAP2A/FOXJ1/EYA1/NPHS2/NPHS1/CER1/FRAS1/SIX2/PROM1 | 15 |
| BP | GO:0071248 | cellular response to metal ion | 12/242 | 190/18670 | 7.265831e-06 | 9.383338e-04 | 8.531434e-04 | MT1H/MT1G/FOSB/CRHBP/MT1X/MT1M/MT2A/SYT9/MT1F/ACER1/TFAP2A/CYP11B2 | 12 |
| BP | GO:0071276 | cellular response to cadmium ion | 6/242 | 37/18670 | 7.401701e-06 | 9.383338e-04 | 8.531434e-04 | MT1H/MT1G/MT1X/MT1M/MT2A/MT1F | 6 |
| BP | GO:0072310 | glomerular epithelial cell development | 4/242 | 11/18670 | 8.460288e-06 | 1.025902e-03 | 9.327613e-04 | ADIPOQ/FOXJ1/NPHS2/NPHS1 | 4 |
| BP | GO:0072009 | nephron epithelium development | 9/242 | 109/18670 | 1.231022e-05 | 1.430550e-03 | 1.300672e-03 | ADIPOQ/WNT7B/FOXD1/FOXJ1/EYA1/NPHS2/NPHS1/SIX2/PROM1 | 9 |
| BP | GO:0072006 | nephron development | 10/242 | 142/18670 | 1.655480e-05 | 1.825802e-03 | 1.660039e-03 | ADIPOQ/BMP7/WNT7B/FOXD1/FOXJ1/EYA1/NPHS2/NPHS1/SIX2/PROM1 | 10 |
| BP | GO:0001822 | kidney development | 14/242 | 278/18670 | 1.702074e-05 | 1.825802e-03 | 1.660039e-03 | ADIPOQ/BMPER/BMP7/WNT7B/FOXD1/TFAP2A/FOXJ1/EYA1/NPHS2/NPHS1/CER1/FRAS1/SIX2/PROM1 | 14 |
| BP | GO:0001867 | complement activation, lectin pathway | 4/242 | 13/18670 | 1.795965e-05 | 1.855165e-03 | 1.686737e-03 | FCN3/FCN2/COLEC10/KRT1 | 4 |
| BP | GO:0035850 | epithelial cell differentiation involved in kidney development | 6/242 | 44/18670 | 2.083444e-05 | 2.003698e-03 | 1.821785e-03 | ADIPOQ/FOXJ1/NPHS2/NPHS1/SIX2/PROM1 | 6 |
| BP | GO:0046688 | response to copper ion | 6/242 | 44/18670 | 2.083444e-05 | 2.003698e-03 | 1.821785e-03 | MT1H/MT1G/MT1X/MT1M/MT2A/MT1F | 6 |
| BP | GO:0010038 | response to metal ion | 16/242 | 364/18670 | 2.306180e-05 | 2.067321e-03 | 1.879631e-03 | MT1H/MT1G/FOSB/TFF1/CRHBP/MT1X/MT1M/MT2A/SYT9/MT1F/ACER1/TFAP2A/TRPV6/CYP11B2/SLC30A2/SLC30A3 | 16 |
| BP | GO:0035107 | appendage morphogenesis | 10/242 | 148/18670 | 2.371971e-05 | 2.067321e-03 | 1.879631e-03 | BMP7/DKK1/HOXD13/TFAP2A/DLX6/COL2A1/FRAS1/FGF4/ALX1/SP8 | 10 |
| BP | GO:0035108 | limb morphogenesis | 10/242 | 148/18670 | 2.371971e-05 | 2.067321e-03 | 1.879631e-03 | BMP7/DKK1/HOXD13/TFAP2A/DLX6/COL2A1/FRAS1/FGF4/ALX1/SP8 | 10 |
| BP | GO:0071241 | cellular response to inorganic substance | 12/242 | 217/18670 | 2.753417e-05 | 2.327054e-03 | 2.115783e-03 | MT1H/MT1G/FOSB/CRHBP/MT1X/MT1M/MT2A/SYT9/MT1F/ACER1/TFAP2A/CYP11B2 | 12 |
| BP | GO:0030326 | embryonic limb morphogenesis | 9/242 | 125/18670 | 3.690323e-05 | 2.940660e-03 | 2.673681e-03 | BMP7/DKK1/HOXD13/TFAP2A/DLX6/FRAS1/FGF4/ALX1/SP8 | 9 |
| BP | GO:0035113 | embryonic appendage morphogenesis | 9/242 | 125/18670 | 3.690323e-05 | 2.940660e-03 | 2.673681e-03 | BMP7/DKK1/HOXD13/TFAP2A/DLX6/FRAS1/FGF4/ALX1/SP8 | 9 |
| BP | GO:0061318 | renal filtration cell differentiation | 4/242 | 17/18670 | 5.738935e-05 | 4.325916e-03 | 3.933170e-03 | ADIPOQ/NPHS2/NPHS1/PROM1 | 4 |
| BP | GO:0072112 | glomerular visceral epithelial cell differentiation | 4/242 | 17/18670 | 5.738935e-05 | 4.325916e-03 | 3.933170e-03 | ADIPOQ/NPHS2/NPHS1/PROM1 | 4 |
| BP | GO:0061005 | cell differentiation involved in kidney development | 6/242 | 54/18670 | 6.841866e-05 | 5.021569e-03 | 4.565666e-03 | ADIPOQ/FOXJ1/NPHS2/NPHS1/SIX2/PROM1 | 6 |
| BP | GO:0090092 | regulation of transmembrane receptor protein serine/threonine kinase signaling pathway | 12/242 | 241/18670 | 7.616192e-05 | 5.245935e-03 | 4.769661e-03 | GDF2/CIDEA/BMP10/BMPER/BMP7/DMRT1/DKK1/FOXD1/CER1/OVOL2/SFRP5/VEPH1 | 12 |
| BP | GO:0071772 | response to BMP | 10/242 | 170/18670 | 7.711844e-05 | 5.245935e-03 | 4.769661e-03 | GDF2/BMP10/BMPER/BMP7/ZCCHC12/DKK1/FOXD1/COL2A1/CER1/SFRP5 | 10 |
| BP | GO:0071773 | cellular response to BMP stimulus | 10/242 | 170/18670 | 7.711844e-05 | 5.245935e-03 | 4.769661e-03 | GDF2/BMP10/BMPER/BMP7/ZCCHC12/DKK1/FOXD1/COL2A1/CER1/SFRP5 | 10 |
| BP | GO:0007586 | digestion | 9/242 | 139/18670 | 8.455653e-05 | 5.614957e-03 | 5.105180e-03 | APOA4/TFF1/GUCA2B/CCKBR/TAC1/SLC9A4/PRSS1/CLPS/SST | 9 |
| BP | GO:0048562 | embryonic organ morphogenesis | 13/242 | 288/18670 | 1.039980e-04 | 6.745356e-03 | 6.132952e-03 | BMP7/HOXA6/TFAP2A/DLX6/HMX2/COL2A1/EYA1/SIX2/SIX3/OVOL2/MYO3A/HMX3/ALX1 | 13 |
| BP | GO:0048736 | appendage development | 10/242 | 179/18670 | 1.183648e-04 | 7.335984e-03 | 6.669958e-03 | BMP7/DKK1/HOXD13/TFAP2A/DLX6/COL2A1/FRAS1/FGF4/ALX1/SP8 | 10 |
| BP | GO:0060173 | limb development | 10/242 | 179/18670 | 1.183648e-04 | 7.335984e-03 | 6.669958e-03 | BMP7/DKK1/HOXD13/TFAP2A/DLX6/COL2A1/FRAS1/FGF4/ALX1/SP8 | 10 |
| BP | GO:0009952 | anterior/posterior pattern specification | 11/242 | 219/18670 | 1.402199e-04 | 8.370288e-03 | 7.610358e-03 | EMX2/DKK1/HOXA6/HOXD13/CER1/SIX2/SIX3/HOXB9/TDRD5/DMRT2/ALX1 | 11 |
| BP | GO:2000826 | regulation of heart morphogenesis | 5/242 | 39/18670 | 1.410554e-04 | 8.370288e-03 | 7.610358e-03 | BMP10/BMP7/DKK1/ISL1/EYA1 | 5 |
| BP | GO:0032835 | glomerulus development | 6/242 | 62/18670 | 1.493873e-04 | 8.676022e-03 | 7.888335e-03 | ADIPOQ/BMP7/FOXJ1/NPHS2/NPHS1/PROM1 | 6 |
| BP | GO:0042471 | ear morphogenesis | 8/242 | 118/18670 | 1.524292e-04 | 8.676022e-03 | 7.888335e-03 | TFAP2A/DLX6/HMX2/COL2A1/EYA1/SIX2/MYO3A/HMX3 | 8 |
| BP | GO:0046686 | response to cadmium ion | 6/242 | 63/18670 | 1.633424e-04 | 9.111238e-03 | 8.284037e-03 | MT1H/MT1G/MT1X/MT1M/MT2A/MT1F | 6 |
