# Supplementary material for: Identification METTL18 as a Potential Prognosis Biomarker and Associated With Immune Infiltrates in Hepatocellular Carcinoma
Source: Front Oncol. 2021 May 26;11:665192. doi: 10.3389/fonc.2021.665192 (PMC8187872; doi:10.3389/fonc.2021.665192)
Supplement: Supplementary Table 4 — The METTL18 protein to protein interactions. [file Table_4.docx]

| node1 | node2 | node1_external_id | node2_external_id | neighborhood_on_chromosome | gene_fusion | phylogenetic_cooccurrence | coexpression | experimentally_determined_interaction | database_annotated | automated_textmining | combined_score |
| --- | --- | --- | --- | --- | --- | --- | --- | --- | --- | --- | --- |
| NPHS1 | NPHS2 | ENSP00000368190 | ENSP00000356587 | 0.000 | 0 | 0.000000 | 0.062 | 0.420 | 0.9 | 0.976000 | 0.998 |
| SERPINB4 | SERPINB3 | ENSP00000343445 | ENSP00000283752 | 0.000 | 0 | 0.007184 | 0.955 | 0.832 | 0.0 | 0.012480 | 0.992 |
| TAC1 | TRH | ENSP00000321106 | ENSP00000303452 | 0.000 | 0 | 0.000000 | 0.061 | 0.000 | 0.9 | 0.709000 | 0.970 |
| EYA1 | SIX2 | ENSP00000342626 | ENSP00000304502 | 0.000 | 0 | 0.000000 | 0.062 | 0.619 | 0.0 | 0.922000 | 0.970 |
| COLEC10 | FCN2 | ENSP00000332723 | ENSP00000291744 | 0.000 | 0 | 0.000000 | 0.062 | 0.157 | 0.9 | 0.640000 | 0.967 |
| COL2A1 | COL9A2 | ENSP00000369889 | ENSP00000361834 | 0.000 | 0 | 0.098470 | 0.340 | 0.379 | 0.9 | 0.162132 | 0.966 |
| COLEC10 | FCN3 | ENSP00000332723 | ENSP00000270879 | 0.000 | 0 | 0.000000 | 0.061 | 0.157 | 0.9 | 0.581000 | 0.962 |
| NPHS1 | KIRREL2 | ENSP00000368190 | ENSP00000353331 | 0.000 | 0 | 0.000000 | 0.000 | 0.348 | 0.9 | 0.314080 | 0.953 |
| GHRH | SST | ENSP00000362716 | ENSP00000287641 | 0.000 | 0 | 0.000000 | 0.000 | 0.000 | 0.0 | 0.952000 | 0.952 |
| PRSS1 | KRT1 | ENSP00000308720 | ENSP00000252244 | 0.000 | 0 | 0.000000 | 0.061 | 0.948 | 0.0 | 0.067000 | 0.950 |
| TAC1 | SST | ENSP00000321106 | ENSP00000287641 | 0.000 | 0 | 0.000000 | 0.190 | 0.000 | 0.0 | 0.932000 | 0.943 |
| SNAP91 | SYT9 | ENSP00000400459 | ENSP00000324419 | 0.000 | 0 | 0.000000 | 0.314 | 0.123 | 0.9 | 0.160000 | 0.942 |
| TAC1 | HTR2C | ENSP00000321106 | ENSP00000276198 | 0.000 | 0 | 0.000000 | 0.111 | 0.000 | 0.9 | 0.367000 | 0.938 |
| CCKBR | TAC1 | ENSP00000335544 | ENSP00000321106 | 0.000 | 0 | 0.000000 | 0.130 | 0.000 | 0.9 | 0.348000 | 0.938 |
| GAD2 | GLS2 | ENSP00000365437 | ENSP00000310447 | 0.076 | 0 | 0.000000 | 0.148 | 0.000 | 0.9 | 0.294000 | 0.937 |
| SAA1 | CXCL5 | ENSP00000384906 | ENSP00000296027 | 0.000 | 0 | 0.000000 | 0.064 | 0.000 | 0.9 | 0.358000 | 0.934 |
| CRHR1 | GHRH | ENSP00000381333 | ENSP00000362716 | 0.000 | 0 | 0.000000 | 0.000 | 0.085 | 0.9 | 0.326000 | 0.932 |
| PAK7 | PAK3 | ENSP00000367686 | ENSP00000353864 | 0.000 | 0 | 0.000000 | 0.064 | 0.177 | 0.9 | 0.102720 | 0.924 |
| CHRM2 | SST | ENSP00000399745 | ENSP00000287641 | 0.000 | 0 | 0.000000 | 0.063 | 0.000 | 0.9 | 0.262000 | 0.924 |
| TRH | HTR2C | ENSP00000303452 | ENSP00000276198 | 0.000 | 0 | 0.000000 | 0.061 | 0.000 | 0.9 | 0.248000 | 0.923 |
| CEACAM5 | CEACAM7 | ENSP00000221992 | ENSP00000006724 | 0.000 | 0 | 0.000000 | 0.213 | 0.000 | 0.9 | 0.029151 | 0.920 |
| CCKBR | TRH | ENSP00000335544 | ENSP00000303452 | 0.000 | 0 | 0.000000 | 0.000 | 0.000 | 0.9 | 0.228000 | 0.919 |
| BMP7 | BMP10 | ENSP00000379204 | ENSP00000295379 | 0.000 | 0 | 0.000000 | 0.000 | 0.000 | 0.9 | 0.181200 | 0.917 |
| CCKBR | HTR2C | ENSP00000335544 | ENSP00000276198 | 0.000 | 0 | 0.000000 | 0.119 | 0.000 | 0.9 | 0.102795 | 0.916 |
| GHRH | TRH | ENSP00000362716 | ENSP00000303452 | 0.000 | 0 | 0.000000 | 0.000 | 0.000 | 0.0 | 0.914000 | 0.915 |
| COL25A1 | COL2A1 | ENSP00000382083 | ENSP00000369889 | 0.000 | 0 | 0.101572 | 0.088 | 0.000 | 0.9 | 0.026800 | 0.915 |
| GGT6 | GAD2 | ENSP00000370962 | ENSP00000365437 | 0.000 | 0 | 0.000000 | 0.062 | 0.000 | 0.9 | 0.160000 | 0.914 |
| MT1G | MT1H | ENSP00000391397 | ENSP00000330587 | 0.000 | 0 | 0.000000 | 0.912 | 0.000 | 0.0 | 0.017469 | 0.913 |
| FCN2 | FCN3 | ENSP00000291744 | ENSP00000270879 | 0.000 | 0 | 0.000000 | 0.099 | 0.000 | 0.9 | 0.060522 | 0.911 |
| COL25A1 | COL9A2 | ENSP00000382083 | ENSP00000361834 | 0.000 | 0 | 0.120350 | 0.000 | 0.000 | 0.9 | 0.000000 | 0.911 |
| CEACAM7 | PRSS21 | ENSP00000006724 | ENSP00000005995 | 0.000 | 0 | 0.000000 | 0.000 | 0.000 | 0.9 | 0.141000 | 0.910 |
| CASP14 | SPRR2A | ENSP00000393417 | ENSP00000376423 | 0.000 | 0 | 0.000000 | 0.062 | 0.000 | 0.9 | 0.118000 | 0.910 |
| GP2 | CEACAM5 | ENSP00000370767 | ENSP00000221992 | 0.000 | 0 | 0.000000 | 0.000 | 0.000 | 0.9 | 0.128000 | 0.909 |
| FGF20 | FGF4 | ENSP00000180166 | ENSP00000168712 | 0.000 | 0 | 0.000000 | 0.000 | 0.000 | 0.9 | 0.092904 | 0.908 |
| TAC1 | PROK1 | ENSP00000321106 | ENSP00000271331 | 0.000 | 0 | 0.000000 | 0.000 | 0.000 | 0.9 | 0.095000 | 0.905 |
| CEACAM5 | PRSS21 | ENSP00000221992 | ENSP00000005995 | 0.000 | 0 | 0.000000 | 0.000 | 0.000 | 0.9 | 0.079000 | 0.904 |
| SNAP91 | CHRM2 | ENSP00000400459 | ENSP00000399745 | 0.000 | 0 | 0.000000 | 0.084 | 0.000 | 0.9 | 0.000000 | 0.904 |
| LYPD2 | PRSS21 | ENSP00000352163 | ENSP00000005995 | 0.000 | 0 | 0.000000 | 0.000 | 0.073 | 0.9 | 0.000000 | 0.903 |
| RXFP4 | SST | ENSP00000357301 | ENSP00000287641 | 0.000 | 0 | 0.000000 | 0.000 | 0.000 | 0.9 | 0.077000 | 0.903 |
| GP2 | PRSS21 | ENSP00000370767 | ENSP00000005995 | 0.000 | 0 | 0.000000 | 0.062 | 0.000 | 0.9 | 0.056000 | 0.903 |
| SAA1 | SST | ENSP00000384906 | ENSP00000287641 | 0.000 | 0 | 0.000000 | 0.000 | 0.000 | 0.9 | 0.079000 | 0.903 |
| CHRM2 | CXCL5 | ENSP00000399745 | ENSP00000296027 | 0.000 | 0 | 0.000000 | 0.000 | 0.000 | 0.9 | 0.079000 | 0.903 |
| CHRM2 | SYT9 | ENSP00000399745 | ENSP00000324419 | 0.000 | 0 | 0.000000 | 0.061 | 0.000 | 0.9 | 0.059000 | 0.903 |
| CXCL5 | SST | ENSP00000296027 | ENSP00000287641 | 0.000 | 0 | 0.000000 | 0.000 | 0.000 | 0.9 | 0.051000 | 0.901 |
| TRH | PROK1 | ENSP00000303452 | ENSP00000271331 | 0.000 | 0 | 0.000000 | 0.055 | 0.000 | 0.9 | 0.000000 | 0.901 |
| HTR2C | PROK1 | ENSP00000276198 | ENSP00000271331 | 0.000 | 0 | 0.000000 | 0.000 | 0.000 | 0.9 | 0.000000 | 0.900 |
| CCKBR | PROK1 | ENSP00000335544 | ENSP00000271331 | 0.000 | 0 | 0.000000 | 0.000 | 0.000 | 0.9 | 0.000000 | 0.900 |
| LYPD2 | CEACAM7 | ENSP00000352163 | ENSP00000006724 | 0.000 | 0 | 0.000000 | 0.000 | 0.000 | 0.9 | 0.000000 | 0.900 |
| LYPD2 | CEACAM5 | ENSP00000352163 | ENSP00000221992 | 0.000 | 0 | 0.000000 | 0.000 | 0.000 | 0.9 | 0.000000 | 0.900 |
| RXFP4 | CXCL5 | ENSP00000357301 | ENSP00000296027 | 0.000 | 0 | 0.000000 | 0.000 | 0.000 | 0.9 | 0.000000 | 0.900 |
