# Supplementary material for: Identification METTL18 as a Potential Prognosis Biomarker and Associated With Immune Infiltrates in Hepatocellular Carcinoma
Source: Front Oncol. 2021 May 26;11:665192. doi: 10.3389/fonc.2021.665192 (PMC8187872; doi:10.3389/fonc.2021.665192)
Supplement: Supplementary Table 5 — METTL18-Gene sets enriched in phenotype high. p. adjusted, adjusted P-value; NES, normalized enrichment score. [file Table_5.docx]

| ID | Set Size | Enrichment score | NES | P value | P adjust | FDR | rank | Leading edge | Core enrichment |
| --- | --- | --- | --- | --- | --- | --- | --- | --- | --- |
| HALLMARK_G2M_CHECKPOINT | 191 | 0.664 | 3.208 | 0.002 | 0.006 | 0.003 | ·8226 | tags=52%, list=16%, signal=44% | HOXC10/EGF/CENPF/NEK2/KIF15/TOP2A/EXO1/KIF11/BUB1/TTK/KIF2C/CENPE/CENPA/CDK1/PLK1/RAD54L/KIF4A/CDKN3/RACGAP1/NDC80/BARD1/TPX2/PBK/POLQ/MCM2/PRC1/CDC20/UBE2C/CCNB2/MKI67/CDC6/PLK4/MYBL2/LMNB1/TROAP/STIL/ORC6/NUSAP1/AURKB/RBL1/HMGA1/MCM6/EFNA5/ESPL1/CHEK1/SMC4/MAD2L1/FBXO5/EZH2/KIF20B/CDC7/HMMR/CBX1/CCNF/RASAL2/INCENP/MCM3/CDC45/UCK2/STMN1/E2F2/KPNA2/MEIS2/TRAIP/CDC25A/E2F3/LBR/SMC2/BIRC5/SLC38A1/PTTG3P/PTTG1/BRCA2/TACC3/DBF4/PRIM2/E2F1/MCM5/CASP8AP2/CDK4/SMARCC1/TGFB1/CDKN2C/POLA2/MTF2/H2AFZ/KPNB1/SMAD3/TFDP1/DKC1/ILF3/HNRNPU/SLC7A1/CHAF1A/HIF1A/CKS1B/PRPF4B/LIG3/DDX39A/TMPO |
| HALLMARK_MYC_TARGETS_V1 | 196 | 0.406 | 1.970 | 0.002 | 0.006 | 0.003 | 14331 | tags=47%, list=28%, signal=34% | MCM2/CDC20/MCM6/MAD2L1/TYMS/CAD/CDC45/KPNA2/MCM4/DEK/CSTF2/ILF2/EPRS/USP1/RRM1/RFC4/MRPL9/MCM5/CDK4/PCNA/SMARCC1/NAP1L1/UBA2/HDAC2/CDK2/MCM7/IARS/HNRNPA1/H2AFZ/KPNB1/TFDP1/NPM1/HNRNPU/XPOT/SRPK1/CBX3/UBE2E1/CCT3/NOP56/SSB/HSP90AB1/CCT5/DHX15/PRPS2/CCT2/TRIM28/ORC2/HNRNPA3/NCBP2/CCNA2/YWHAQ/VBP1/SRSF3/BUB3/PABPC1/LSM2/EIF3D/RAN/SET/HNRNPR/DDX18/EIF4G2/SRSF7/XPO1/SRSF1/HNRNPC/SF3B3/SNRPB2/RNPS1/HNRNPA2B1/HDGF/HNRNPD/CANX/HDDC2/CCT4/PTGES3/SNRPD1/PGK1/FBL/GLO1/TRA2B/NOLC1/DDX21/SRSF2/APEX1/PABPC4/NHP2/SF3A1/PWP1/POLE3/XRCC6/ERH |
| HALLMARK_E2F_TARGETS | 197 | 0.673 | 3.264 | 0.002 | 0.006 | 0.003 | 9997 | tags=62%, list=20%, signal=50% | TOP2A/BUB1B/KIF18B/DLGAP5/TRIP13/KIF2C/CENPE/CDK1/PLK1/DEPDC1/KIF4A/HELLS/GINS1/CDCA8/CDKN3/RACGAP1/BARD1/MELK/UBE2T/E2F8/MCM2/RAD51AP1/CDC20/CCNB2/MKI67/PLK4/NCAPD2/MYBL2/LMNB1/ORC6/TCF19/AURKB/GINS4/CIT/HMGA1/SPC25/MCM6/ESPL1/CHEK1/ASF1B/SMC4/MAD2L1/MMS22L/PSMC3IP/MSH2/EZH2/BRCA1/HMMR/MCM3/CDCA3/STMN1/KPNA2/DSCC1/MCM4/CDC25A/SMC3/LBR/DEK/BIRC5/HMGB2/PTTG1/BRCA2/TACC3/DNMT1/USP1/ANP32E/PRIM2/CENPM/POLD1/RRM2/NUP205/MCM5/RFC3/CDK4/PCNA/NUP153/NAP1L1/SPC24/PSIP1/DCK/TIMELESS/LIG1/NUP107/CDKN2C/MCM7/POLA2/PRKDC/GINS3/H2AFZ/RPA1/DCLRE1B/POLD3/RAD1/TP53/EED/TUBB/ILF3/SSRP1/CKS1B/ATAD2/CDKN2A/CSE1L/IPO7/TIPIN/DDX39A/SPAG5/TMPO/CCP110/H2AFX/SNRPB/NOP56/STAG1/CBX5/PMS2/RNASEH2A/DONSON/SLBP/ORC2/LUC7L3/CKS2/MXD3/RAD21/RBBP7 |
| HALLMARK_KRAS_SIGNALING_DN | 193 | 0.474 | 2.289 | 0.002 | 0.006 | 0.003 | 3893 | tags=25%, list=8%, signal=24% | CPA2/BRDT/CLPS/SLC6A14/SLC30A3/NPHS1/COL2A1/CYP11B2/TEX15/KLK8/SCGB1A1/KLK7/PAX3/GRID2/TFAP2B/SHOX2/CACNG1/P2RY4/ADRA2C/EGF/KRT4/NGB/CDH16/HTR1D/KLHDC8A/RYR2/EPHA5/RIBC2/ATP4A/TGFB2/DLK2/BARD1/NUDT11/NPY4R/CD207/ALOX12B/CKM/H2AFY2/TNNI3/PLAG1/ZNF112/MYH7/FGFR3/CAPN9/YBX2/GPR19/MSH5/AKR1B10/TFCP2L1 |
| HALLMARK_MITOTIC_SPINDLE | 195 | 0.565 | 2.735 | 0.002 | 0.006 | 0.003 | 13018 | tags=53%, list=26%, signal=40% | KIF3C/CENPF/ANLN/NEK2/KIF15/TOP2A/KIF11/BUB1/TTK/DLGAP5/KIF2C/CENPE/CDK1/PLK1/KIF4A/RACGAP1/NDC80/ECT2/TPX2/PRC1/CCNB2/KNTC1/LMNB1/NUSAP1/ARHGEF3/ESPL1/SMC4/FBXO5/NCK2/KIF20B/WASF1/ARHGEF2/RASAL2/INCENP/ARHGEF11/SASS6/FSCN1/FGD6/CENPJ/SMC3/PIF1/CNTRL/BIRC5/CD2AP/BRCA2/FLNA/ALMS1/CEP72/LLGL1/MAPRE1/CEP250/KIFAP3/CKAP5/NIN/CLIP2/CCDC88A/BCL2L11/TUBD1/MID1IP1/CEP192/CDC42BPA/SOS1/KIF5B/ABL1/TSC1/FARP1/RASA2/VCL/CYTH2/CDC27/LRPPRC/LATS1/ARFGEF1/RASA1/TUBGCP3/WASF2/EPB41L2/ABI1/DYNC1H1/PALLD/ARHGAP27/TRIO/PLEKHG2/CDC42EP4/RHOF/PKD2/MAP1S/SMC1A/RALBP1/RICTOR/ACTN4/SSH2/CNTROB/HOOK3/RABGAP1/RAB3GAP1/CTTN/TLK1/GEMIN4/NOTCH2/NUMA1/KATNA1/OPHN1/NF1 |
| HALLMARK_SPERMATOGENESIS | 134 | 0.450 | 2.076 | 0.002 | 0.006 | 0.003 | 8515 | tags=38%, list=17%, signal=32% | CRISP2/SNAP91/TKTL1/DMRT1/TUBA3C/NEK2/NCAPH/BUB1/PGK2/TTK/KIF2C/CDK1/NAA11/CDKN3/CLGN/CFTR/PDHA2/DDX4/CCNB2/SLC2A5/CHRM4/TNNI3/GFI1/SYCP1/EZH2/TNP1/GAD1/YBX2/NPHP1/RPL39L/ACRV1/HIST1H1T/MTNR1A/ADAM2/DBF4/TOPBP1/RFC4/TEKT2/HSPA1L/GSTM3/DMC1/ELOVL3/COIL/STRBP/HTR5A/CNIH2/MLF1/ARL4A/GAPDHS/CCNA1/ART3 |
| HALLMARK_COAGULATION | 137 | -0.477 | -2.221 | 0.002 | 0.006 | 0.003 | 5912 | tags=43%, list=12%, signal=38% | MSRB2/CTSO/FBN1/PLAT/CRIP2/CFD/SERPINC1/FURIN/TMPRSS6/GDA/THBS1/PROS1/TIMP3/SERPINA1/RAPGEF3/RGN/HPN/CLU/PROC/THBD/GSN/F2/C3/F10/FYN/HTRA1/CFB/CPB2/ITIH1/ACOX2/C1S/HMGCS2/MBL2/TF/MASP2/APOC1/VWF/CFI/SERPING1/C8G/C8B/FGG/HRG/F12/MST1/C1R/KLKB1/PLG/F11/FGA/ANG/APOA1/GP9/APOC3/PROZ/C8A/C9/PF4/F9 |
| HALLMARK_BILE_ACID_METABOLISM | 112 | -0.578 | -2.597 | 0.002 | 0.006 | 0.003 | 9309 | tags=49%, list=18%, signal=40% | KLF1/HSD3B7/RXRA/ABCG8/CROT/CYP27A1/NR0B2/LIPE/PRDX5/RBP1/PAOX/GSTK1/SLCO1A2/HACL1/NR3C2/SERPINA6/PHYH/SLC22A18/FADS2/DIO1/IDH2/AR/SOD1/GC/LONP2/CYP7A1/ABCA9/CYP46A1/FDXR/CAT/ABCA6/MLYCD/PXMP2/PIPOX/GNMT/SCP2/SULT1B1/PEX11G/EPHX2/HAO1/AGXT/ACSL1/HSD17B6/NR1I2/TTR/APOA1/SLC27A2/ALDH8A1/BBOX1/CYP39A1/ABCA8/SLC27A5/CYP8B1/AKR1D1/DIO2 |
| HALLMARK_ANDROGEN_RESPONSE | 96 | -0.407 | -1.775 | 0.002 | 0.006 | 0.003 | 2547 | tags=24%, list=5%, signal=23% | SPCS3/TMPRSS2/DNAJB9/DBI/ELL2/SORD/MAF/SGK1/B2M/NKX3-1/LIFR/SLC38A2/ABHD2/SAT1/HSD17B14/HPGD/ADAMTS1/INSIG1/STEAP4/AZGP1/AKAP12/KLK2/KLK3 |
| HALLMARK_TNFA_SIGNALING_VIA_NFKB | 195 | -0.426 | -2.072 | 0.002 | 0.006 | 0.003 | 5903 | tags=26%, list=12%, signal=23% | SPSB1/CD80/KLF6/SGK1/PNRC1/BHLHE40/SOD2/KLF9/GPR183/SDC4/KLF2/MAP2K3/ID2/TRIB1/EGR2/DUSP2/ETS2/CEBPB/SERPINB8/PHLDA1/EGR1/CD69/GEM/NFIL3/SAT1/NAMPT/SOCS3/RHOB/ZFP36/PDE4B/FOSL1/G0S2/CEBPD/SIK1/GADD45A/ATF3/CXCL2/PTGS2/RCAN1/EGR3/DUSP1/GCH1/GADD45B/AREG/ACKR3/NR4A1/NR4A2/FOS/IL6/NR4A3/FOSB |
| HALLMARK_WNT_BETA_CATENIN_SIGNALING | 42 | 0.567 | 2.079 | 0.002 | 0.006 | 0.003 | 9134 | tags=50%, list=18%, signal=41% | DKK1/DKK4/AXIN2/WNT6/NKD1/FZD1/JAG1/LEF1/DVL2/GNAI1/TCF7/SKP2/PTCH1/ADAM17/HDAC2/MAML1/TP53/NCSTN/NOTCH1/KAT2A/HDAC11 |
| HALLMARK_ADIPOGENESIS | 194 | -0.508 | -2.468 | 0.002 | 0.006 | 0.003 | 7985 | tags=41%, list=16%, signal=34% | LIPE/COX6A1/SLC25A1/NDUFAB1/SORBS1/MARC2/DHRS7B/AIFM1/TOB1/ECH1/ADIG/GPX4/UQCR10/SUCLG1/DBT/UQCRC1/MYLK/HIBCH/CMBL/PHYH/GRPEL1/GPHN/NDUFA5/LIFR/COX7B/CRAT/PEX14/LPL/COL15A1/HADH/NDUFB7/SOD1/STOM/ITGA7/FZD4/PGM1/COQ9/CD302/POR/UQCRQ/GPX3/PTGER3/C3/TST/CHCHD10/SDHB/DDT/CAT/ETFB/ACADM/DECR1/UQCR11/SULT1A1/PLIN2/CPT2/CYP4B1/PEMT/SLC25A10/ECHS1/FAH/QDPR/APOE/ACADS/ACADL/REEP6/ACOX1/SCP2/GADD45A/ACAA2/ALDH2/ORM1/EPHX2/ANGPTL4/OMD/MRAP/LEP/FABP4/CIDEA/ADIPOQ |
| HALLMARK_INTERFERON_GAMMA_RESPONSE | 199 | -0.401 | -1.952 | 0.002 | 0.006 | 0.003 | 7316 | tags=23%, list=14%, signal=19% | GZMA/CFH/FGL2/UPP1/OAS2/UBE2L6/MX1/IL18BP/HLA-B/IFI44/IFITM2/CXCL9/SOD2/PSMA2/B2M/IRF8/LY6E/APOL6/LAP3/VAMP5/METTL7B/P2RY14/FAS/TNFSF10/CD69/CFB/KLRK1/NAMPT/SOCS3/C1S/PDE4B/ISG20/IFI44L/BST2/OASL/SERPING1/SELP/PTGS2/C1R/ISG15/GCH1/IRF4/IFI27/IL6/MT2A |
| HALLMARK_XENOBIOTIC_METABOLISM | 199 | -0.594 | -2.895 | 0.002 | 0.006 | 0.003 | 8125 | tags=55%, list=16%, signal=46% | MAN1A1/COMT/KYNU/SLC12A4/MCCC2/PTS/CBR1/TMBIM6/ECH1/UPP1/CNDP2/ACP2/TTPA/ACSM1/GSTT2/GSTO1/AOX1/PAPSS2/HACL1/PGRMC1/SERPINA6/LPIN2/CYP2E1/IRF8/CES1/ABHD6/BLVRB/UPB1/ID2/PROS1/VTN/ALAS1/HSD17B2/EPHA2/FAS/TMEM176B/SLC35D1/ITIH4/ETS2/POR/SERTAD1/CYB5A/SLC6A12/CDO1/CSAD/CYP17A1/DDT/F10/CAT/G6PC/CYP2J2/IGF1/CFB/PINK1/ATOH8/PEMT/NDRG2/FAH/GCKR/ITIH1/GNMT/GABARAPL1/APOE/ACOX2/CYFIP2/IGFBP1/MBL2/MTHFD1/ACOX1/DHRS1/CA2/ANGPTL3/PC/ADH1C/VNN1/SLC46A3/ETFDH/IGFBP4/PDK4/ALDH2/CDA/HSD11B1/HRG/ARG2/ENPEP/CYP4F2/HGFAC/XDH/PLG/F11/FETUB/LEAP2/CYP2C18/ASL/ARG1/TDO2/CRP/RBP4/GCH1/FABP1/LCAT/ESR1/DCXR/CYP1A2/FBP1/CYP26A1/TAT/SLC22A1/MT2A |
| HALLMARK_OXIDATIVE_PHOSPHORYLATION | 184 | -0.505 | -2.445 | 0.002 | 0.006 | 0.003 | 13044 | tags=50%, list=26%, signal=37% | NDUFA8/COX7C/OGDH/DLD/MDH2/DLST/COX8A/BCKDHA/FXN/UQCRFS1/TIMM8B/NDUFC2/RETSAT/MRPS12/UQCRC2/NDUFS3/CYCS/ATP6V0B/CPT1A/UQCRB/NNT/NDUFA2/LDHB/COX7A2/TIMM13/MAOB/HADHB/COX6A1/NDUFAB1/AIFM1/ECH1/MRPS15/NDUFS6/NDUFA4/SLC25A11/GPX4/NDUFB8/UQCR10/NDUFB2/SUCLG1/NDUFV1/UQCRC1/FDX1/NDUFS7/PMPCA/SDHD/ECI1/PHYH/GRPEL1/COX5A/ACADVL/NDUFA5/SURF1/COX7B/NDUFS8/COX6C/COX5B/NDUFA7/ACADSB/IDH2/NDUFB7/ALAS1/NDUFV2/SLC25A4/SDHA/NDUFB1/NDUFA3/MRPL34/POR/CYB5A/UQCRQ/COX6B1/SLC25A20/HSD17B10/ETFA/SDHB/ETFB/ACADM/DECR1/UQCR11/GOT2/BDH2/ECHS1/ACAT1/COX4I1/ALDH6A1/ETFDH/PDK4/ACAA2/MPC1/ACAA1/OAT |
| HALLMARK_FATTY_ACID_METABOLISM | 157 | -0.526 | -2.490 | 0.002 | 0.006 | 0.003 | 8019 | tags=42%, list=16%, signal=35% | UROD/HADHB/CRYZ/PTS/CBR1/ECH1/PPARA/PCBD1/UBE2L6/XIST/NTHL1/SUCLG1/MCEE/ACOT2/SDHD/ECI1/HIBCH/ACADVL/CRAT/CD1D/HADH/AOC3/SDHA/TP53INP2/HSD17B10/AQP7/SUCLG2/ACADM/DECR1/BCKDHB/CPT2/MLYCD/GRHPR/ECHS1/GABARAPL1/ACADS/HMGCS2/ACADL/G0S2/REEP6/GCDH/ACOX1/CA2/ADH1C/HMGCL/VNN1/ETFDH/HPGD/ACSM3/ACAA2/EHHADH/ACAA1/ACSL1/TDO2/CYP4A11/AADAT/INMT/FABP1/CYP4A22/RDH16/GSTZ1/CA4/GPD1/HAO2/ENO3/CIDEA |
| HALLMARK_HYPOXIA | 190 | -0.380 | -1.841 | 0.002 | 0.006 | 0.003 | 6056 | tags=26%, list=12%, signal=23% | GALK1/ALDOC/SELENBP1/KLF6/PNRC1/BHLHE40/SLC37A4/LALBA/SDC4/FAM162A/ILVBL/DDIT4/UGP2/RORA/ERRFI1/GLRX/PGM1/PKP1/STC1/EDN2/HAS1/PLIN2/NFIL3/TMEM45A/GRHPR/CP/TGM2/ZFP36/IGFBP1/ISG20/DCN/ATF3/NCAN/CA12/SRPX/ANGPTL4/DUSP1/ACKR3/PPARGC1A/ALDOB/GCK/AKAP12/FOS/FBP1/IL6/PCK1/MT1E/ENO3/MT2A |
| HALLMARK_APOPTOSIS | 160 | -0.402 | -1.904 | 0.002 | 0.006 | 0.003 | 6483 | tags=24%, list=13%, signal=21% | MMP2/GPX4/PRF1/PLAT/SOD2/GPX1/TGFBR3/EBP/TIMP3/SOD1/CLU/HGF/FAS/TNFSF10/EREG/ERBB2/GSN/GPX3/MGMT/SC5D/F2/FDXR/CD69/SAT1/RHOB/ISG20/DCN/GADD45A/CD14/ATF3/EMP1/EGR3/GSTM1/GCH1/GADD45B/CTH/AVPR1A/IL6/BCL2L10 |
| HALLMARK_MYOGENESIS | 198 | -0.354 | -1.727 | 0.004 | 0.011 | 0.005 | 5833 | tags=27%, list=12%, signal=24% | FKBP1B/BHLHE40/MYLK/MYOM1/HSPB2/TAGLN/CFD/CRAT/OCEL1/COL15A1/SH3BGR/FHL1/ITGA7/EFS/AK1/CLU/MYL4/IGFBP7/MYH11/MYLPF/GSN/GPX3/SGCA/MYH2/AGL/IGF1/PTGIS/TNNC2/LAMA2/CKB/AEBP1/COX7A1/PC/TNNT3/TCAP/CASQ2/COX6A2/SGCD/MYH3/ACSL1/MYH8/ACTC1/CAMK2B/GADD45B/CKMT2/DES/FXYD1/VIPR1/BDKRB2/PVALB/NOS1/GNAO1/MYH4/ENO3 |
| HALLMARK_PEROXISOME | 104 | -0.382 | -1.692 | 0.006 | 0.015 | 0.006 | 5233 | tags=27%, list=10%, signal=24% | GSTK1/SOD2/SERPINA6/DIO1/CRAT/PEX14/IDH2/SOD1/FIS1/LONP2/SLC25A4/DHRS3/CAT/MLYCD/STS/ACOX1/SCP2/HMGCL/ALB/EHHADH/EPHX2/ABCB4/ACAA1/ACSL1/NR1I2/TTR/SLC27A2/HAO2 |
| HALLMARK_APICAL_JUNCTION | 193 | 0.311 | 1.504 | 0.008 | 0.018 | 0.008 | 4230 | tags=14%, list=8%, signal=13% | SLC30A3/KCNH2/FLNC/CLDN18/AMH/CLDN4/ITGB4/ITGA3/MAPK13/ITGA2/LAMC2/DMP1/DSC3/SRC/CDH6/SGCE/SPEG/ADAM23/ADAM9/GRB7/CALB2/TNFRSF11B/MPZL1/PPP2R2C/ACTA1/FSCN1/GNAI1 |
| HALLMARK_INTERFERON_ALPHA_RESPONSE | 95 | -0.375 | -1.632 | 0.008 | 0.018 | 0.008 | 8137 | tags=21%, list=16%, signal=18% | DHX58/UBA7/EPSTI1/UBE2L6/MX1/IFI44/IFITM2/B2M/GMPR/LY6E/LAP3/TMEM140/C1S/ISG20/IFI44L/BST2/OASL/ISG15/IFITM1/IFI27 |
| HALLMARK_EPITHELIAL_MESENCHYMAL_TRANSITION | 195 | -0.295 | -1.438 | 0.010 | 0.022 | 0.009 | 5654 | tags=25%, list=11%, signal=22% | FBN1/ECM2/LRRC15/MYLK/ACTA2/SLIT3/TAGLN/IGFBP2/SDC4/EFEMP2/COL5A3/TGFBR3/MYL9/ID2/THBS1/IL32/TIMP3/LOXL1/SDC1/CRLF1/FAS/SFRP4/VCAN/FUCA1/GEM/HTRA1/ELN/SAT1/ABI3BP/CXCL12/TGM2/LAMA2/RHOB/GAS1/MFAP5/PCOLCE/DCN/GADD45A/IGFBP4/SGCD/FBLN5/SFRP1/FBLN2/GADD45B/AREG/NNMT/IL6/BASP1 |
| HALLMARK_ESTROGEN_RESPONSE_LATE | 196 | -0.297 | -1.445 | 0.010 | 0.022 | 0.009 | 6035 | tags=23%, list=12%, signal=21% | TSPAN13/PKP3/CPE/AMFR/SGK1/HSPA4L/PAPSS2/SERPINA3/CYP4F11/BLVRB/COX6C/ID2/IDH2/SERPINA1/DUSP2/CISH/PTGER3/TST/ETFB/ABHD2/CXCL12/CKB/TIAM1/ACOX2/ZFP36/HMGCS2/PGR/CXCL14/ISG20/CA2/RPS6KA2/IGFBP4/CA12/EGR3/SLC27A2/TH/TPSAB1/AREG/DCXR/GAL/ASS1/NPY1R/FOS/ASCL1/CHST8/TFF1 |
| HALLMARK_CHOLESTEROL_HOMEOSTASIS | 74 | -0.410 | -1.704 | 0.012 | 0.023 | 0.010 | 5839 | tags=26%, list=12%, signal=23% | ALDOC/PNRC1/LSS/ETHE1/FADS2/EBP/LPL/ERRFI1/CLU/GLDC/SC5D/MVD/NFIL3/ACSS2/ATF3/TM7SF2/CBS/ADH4/AVPR1A |
| HALLMARK_IL2_STAT5_SIGNALING | 194 | -0.289 | -1.403 | 0.013 | 0.023 | 0.010 | 7175 | tags=22%, list=14%, signal=19% | FGL2/SLC2A3/ENPP1/GPX4/CTSZ/KLF6/BHLHE40/GSTO1/RGS16/CAPN3/IRF8/SERPINC1/AKAP2/FURIN/CD81/PTH1R/TNFSF11/CST7/RORA/IL10/TNFSF10/DHRS3/IL18R1/CISH/PHLDA1/PLIN2/NFIL3/SOCS2/FAH/TGM2/GATA1/RHOB/GABARAPL1/TIAM1/TNFRSF1B/CA2/EMP1/SELP/IRF4/GADD45B/PENK/ENO3/IL1RL1 |
| HALLMARK_COMPLEMENT | 197 | -0.292 | -1.424 | 0.013 | 0.023 | 0.010 | 9378 | tags=24%, list=19%, signal=20% | TIMP1/C1QA/DUSP5/CASP9/SPOCK2/KYNU/ATOX1/CSRP1/GZMA/CFH/PRSS36/GZMB/F7/CTSO/PLAT/FDX1/MSRB1/NOTCH4/CPM/SERPINC1/LAP3/TMPRSS6/SERPINA1/CLU/ZFPM2/CEBPB/F2/C3/F10/FYN/CFB/CP/ITIH1/C1S/CA2/APOC1/S100A12/SERPING1/CDA/C1R/KLKB1/PLG/ANG/GP9/C9/IL6/APOA4 |
| HALLMARK_ANGIOGENESIS | 36 | -0.447 | -1.582 | 0.028 | 0.049 | 0.021 | 4245 | tags=31%, list=8%, signal=28% | LPL/VTN/THBD/VCAN/KCNJ8/STC1/APOH/PRG2/SLCO2A1/PGLYRP1/PF4 |
| HALLMARK_PANCREAS_BETA_CELLS | 40 | 0.462 | 1.672 | 0.029 | 0.049 | 0.021 | 3425 | tags=30%, list=7%, signal=28% | SST/ISL1/CHGA/PDX1/NKX6-1/NKX2-2/NEUROD1/GCG/SYT13/NEUROG3/ABCC8/INSM1 |
| HALLMARK_INFLAMMATORY_RESPONSE | 196 | -0.273 | -1.327 | 0.029 | 0.049 | 0.021 | 7215 | tags=21%, list=14%, signal=18% | CCR7/KCNA3/APLNR/SCARF1/CALCRL/SLC4A4/CCL24/KLF6/CXCL9/SLC31A1/SLC31A2/RGS16/IL18RAP/GPR183/LY6E/C5AR1/PCDH7/PTGIR/TACR1/IL10/HPN/TNFSF10/EREG/IL18R1/SLC1A2/CD69/NAMPT/BDKRB1/PDE4B/TNFRSF1B/BST2/CD14/SELE/NMUR1/IFITM1/SLC7A2/GCH1/ROS1/CSF3/IL6/MARCO |
