# Supplementary material for: Identification METTL18 as a Potential Prognosis Biomarker and Associated With Immune Infiltrates in Hepatocellular Carcinoma
Source: Front Oncol. 2021 May 26;11:665192. doi: 10.3389/fonc.2021.665192 (PMC8187872; doi:10.3389/fonc.2021.665192)
Supplement: Supplementary Table 6 — Univariate and multivariate survival results (Disease Specific Survival) of prognostic covariates in HCC patients. [file Table_6.docx]

| Characteristics | Total(N) | HR(95% CI) Univariate analysis | P value Univariate analysis | HR(95% CI) Multivariate analysis | P value Multivariate analysis |
| --- | --- | --- | --- | --- | --- |
| T stage (T1 vs. T2&T3&T4) | 359 | 0.356(0.220-0.577) | <0.001 | 0.594(0.247-1.427) | 0.244 |
| N stage (N0 vs. N1) | 251 | 0.281(0.068-1.165) | 0.080 | 0.850(0.084-8.550) | 0.890 |
| M stage (M0 vs. M1) | 266 | 0.196(0.047-0.813) | 0.025 | 0.273(0.051-1.476) | 0.132 |
| Histologic grade (G1&G2 vs. G4&G3) | 357 | 0.894(0.562-1.422) | 0.636 |  |  |
| Vascular invasion (No vs. Yes) | 306 | 0.779(0.431-1.408) | 0.408 |  |  |
| Residual tumor (R0 vs. R1&R2) | 334 | 0.610(0.264-1.406) | 0.246 |  |  |
| Tumor status (Tumor free vs. With tumor) | 351 | 0.000(0.000-Inf) | 0.994 |  |  |
| Albumin(g/dl) (<3.5 vs. >=3.5) | 291 | 0.846(0.432-1.656) | 0.625 |  |  |
| AFP(ng/ml) (<=400 vs. >400) | 273 | 1.177(0.612-2.265) | 0.625 |  |  |
| TP53 status (WT vs. Mut) | 349 | 0.604(0.370-0.983) | 0.043 | 0.375(0.119-1.185) | 0.095 |
| Child-Pugh grade (A vs. B&C) | 233 | 0.398(0.175-0.907) | 0.028 | 0.335(0.102-1.101) | 0.072 |
| Race (Asian&Black or African American vs. White) | 350 | 0.658(0.410-1.055) | 0.083 | 0.333(0.087-1.276) | 0.109 |
| Adjacent hepatic tissue inflammation (None vs. Mild&Severe) | 229 | 0.691(0.378-1.264) | 0.231 |  |  |
| Age (<=60 vs. >60) | 362 | 1.136(0.730-1.768) | 0.573 |  |  |
| Gender (Female vs. Male) | 362 | 1.190(0.756-1.875) | 0.452 |  |  |
| Prothrombin time (<=4 vs. >4) | 287 | 0.564(0.335-0.952) | 0.032 | 0.948(0.270-3.326) | 0.933 |
| METTL18 (High vs. Low) | 362 | 1.756(1.117-2.761) | 0.015 | 2.488(1.026-6.035) | 0.044 |
