# Supplementary material for: Identification METTL18 as a Potential Prognosis Biomarker and Associated With Immune Infiltrates in Hepatocellular Carcinoma
Source: Front Oncol. 2021 May 26;11:665192. doi: 10.3389/fonc.2021.665192 (PMC8187872; doi:10.3389/fonc.2021.665192)
Supplement: Supplementary Table 7 — Univariate and multivariate survival results (Progression Free Interval) of prognostic covariates in HCC patients. [file Table_7.docx]

| Characteristics | Total(N) | HR (95% CI) Univariate analysis | P value Univariate analysis | HR (95% CI) Multivariate analysis | P value Multivariate analysis |
| --- | --- | --- | --- | --- | --- |
| T stage (T1 vs. T2&T3&T4) | 367 | 0.419(0.309-0.569) | <0.001 | 0.754(0.465-1.222) | 0.251 |
| N stage (N0 vs. N1) | 256 | 0.722(0.178-2.926) | 0.648 |  |  |
| M stage (M0 vs. M1) | 270 | 0.291(0.091-0.926) | 0.037 | 0.382(0.116-1.263) | 0.115 |
| Histologic grade (G1&G2 vs. G4&G3) | 365 | 0.866(0.639-1.174) | 0.354 |  |  |
| Vascular invasion (No vs. Yes) | 314 | 0.595(0.423-0.836) | 0.003 | 0.864(0.536-1.394) | 0.550 |
| Residual tumor (R0 vs. R1&R2) | 341 | 0.660(0.366-1.189) | 0.167 |  |  |
| Albumin(g/dl) (<3.5 vs. >=3.5) | 296 | 1.110(0.753-1.637) | 0.599 |  |  |
| AFP(ng/ml) (<=400 vs. >400) | 277 | 0.947(0.633-1.419) | 0.793 |  |  |
| TP53 status (WT vs. Mut) | 357 | 0.775(0.563-1.068) | 0.119 |  |  |
| Child-Pugh grade (A vs. B&C) | 238 | 0.720(0.395-1.314) | 0.285 |  |  |
| Race (Asian&Black or African American vs. White) | 358 | 0.753(0.558-1.016) | 0.063 | 0.637(0.419-0.968) | 0.035 |
| Adjacent hepatic tissue inflammation (None vs. Mild&Severe) | 233 | 0.808(0.563-1.159) | 0.246 |  |  |
| Age (<=60 vs. >60) | 370 | 1.051(0.784-1.409) | 0.740 |  |  |
| Gender (Female vs. Male) | 370 | 1.028(0.754-1.402) | 0.861 |  |  |
| Prothrombin time (<=4 vs. >4) | 293 | 0.908(0.646-1.277) | 0.579 |  |  |
| METTL18 (High vs. Low) | 370 | 1.510(1.124-2.028) | 0.006 | 1.524(1.012-2.296) | 0.044 |
