# Supplementary material for: Identification METTL18 as a Potential Prognosis Biomarker and Associated With Immune Infiltrates in Hepatocellular Carcinoma
Source: Front Oncol. 2021 May 26;11:665192. doi: 10.3389/fonc.2021.665192 (PMC8187872; doi:10.3389/fonc.2021.665192)
Supplement: Supplementary Table 8 — The prognostic value of METTL18 (Disease Specific Survival) in various HCC subgroups. [file Table_8.docx]

| Characteristics | N (%) | HR (95% CI) | P value |
| --- | --- | --- | --- |
| T stage |  |  |  |
| T1 | 178 (50) | 1.561(0.698-3.492) | 0.278 |
| T2 | 91 (25) | 2.014(0.709-5.723) | 0.189 |
| T3&T4 | 90 (25) | 1.916(0.967-3.798) | 0.062 |
| N stage |  |  |  |
| N0 | 247 (98) | 2.090(1.157-3.773) | 0.015 |
| N1 | 4 (2) | - | - |
| M stage |  |  |  |
| M0 | 263 (99) | 2.369(1.306-4.297) | 0.005 |
| M1 | 3 (1) | - | - |
| Pathologic stage |  |  |  |
| Stage I | 168 (50) | 1.535(0.636-3.706) | 0.340 |
| Stage II | 83 (25) | 1.426(0.466-4.363) | 0.534 |
| Stage III&Stage IV | 87 (26) | 2.126(1.021-4.424) | 0.044 |
| Histologic grade |  |  |  |
| G1 | 55 (15) | 1.346(0.386-4.699) | 0.641 |
| G2 | 171 (48) | 1.375(0.700-2.700) | 0.355 |
| G4&G3 | 131 (37) | 2.560(1.041-6.294) | 0.041 |
