# Supplementary material for: Identification METTL18 as a Potential Prognosis Biomarker and Associated With Immune Infiltrates in Hepatocellular Carcinoma
Source: Front Oncol. 2021 May 26;11:665192. doi: 10.3389/fonc.2021.665192 (PMC8187872; doi:10.3389/fonc.2021.665192)
Supplement: Supplementary Table 9 — The prognostic value of METTL18 (Progression Free Interva) in various HCC subgroups. [file Table_9.docx]

| Characteristics | N (%) | HR (95% CI) | P value |
| --- | --- | --- | --- |
| T stage |  |  |  |
| T1 | 181 (49) | 1.500(0.926-2.432) | 0.100 |
| T2 | 93 (25) | 1.562(0.886-2.753) | 0.123 |
| T3&T4 | 93 (25) | 1.110(0.660-1.865) | 0.694 |
| N stage |  |  |  |
| N0 | 252 (98) | 1.469(1.026-2.104) | 0.036 |
| N1 | 4 (2) | - | - |
| M stage |  |  |  |
| M0 | 266 (99) | 1.568(1.099-2.236) | 0.013 |
| M1 | 4 (1) | - | - |
| Pathologic stage |  |  |  |
| Stage I | 171 (49) | 1.471(0.895-2.417) | 0.127 |
| Stage II | 85 (25) | 1.368(0.751-2.493) | 0.306 |
| Stage III&Stage IV | 90 (26) | 1.151(0.675-1.963) | 0.606 |
| Histologic grade |  |  |  |
| G1 | 55 (15) | 2.017(0.873-4.661) | 0.101 |
| G2 | 177 (48) | 1.225(0.787-1.907) | 0.368 |
| G4&G3 | 133 (36) | 1.780(1.037-3.057) | 0.037 |
